# GRB7 plays a promoting role in the progression of gastric cancer

**DOI:** 10.1186/s12885-023-11694-5

**Published:** 2023-12-21

**Authors:** Guomin Zhu, Hu Cai, Qiang Xiao, Shukun Zeng, Xiaohua Jiang, Donglan Liu

**Affiliations:** 1 Department of General Surgery, The First Affiliated Hospital, Jiangxi Medical College, Nanchang University, Nanchang, 330006 China; 2 Department of General Surgery, The Affiliated Hospital of JiangXi University of Chinese Medicine, Nanchang, 330006 China; 3 Department of Gastroenterology, Cancer Hospital of Jiangxi Province, Jiangxi, 330029 China

**Keywords:** Gastric cancer, GRB7, Molecular target, Cell proliferation

## Abstract

**Background:**

Gastric cancer is a clinically common tumor, showing an upward trend of both incidence and mortality. GRB7 has been identified as a vital regulator in tumor progression. This study aims to uncover the biological function of GRB7 in gastric cancer process.

**Methods:**

immunohistochemical (IHC) staining using a tissue microarray (TMA), quantitative reverse transcription PCR (qRT-PCR) and Western blotting were performed to detect the expression of genes. Furthermore, gastric cancer cell lines AGS and MGC-803 were transfected with short hairpin RNAs against GRB7. The biological function of GRB7 in gastric cancer cells were examined by CCK-8, flow cytometry, wound healing and Transwell assays. Then, in vivo tumor formation assay was conducted to explore the effects of GRB7 on tumor growth. Finally, expression levels of proteins related to cell functions were determined by Western blotting. Coimmunoprecipitation (CoIP) assay was performed to assess the protein-protein interaction.

**Results:**

GRB7 was up-regulated in gastric cancer tissues and cell lines, and its expression was inversely proportional to survival of gastric cancer patients. Moreover, GRB7 knockdown inhibited proliferative, migratory abilities, as well as promoted cell apoptosis in gastric cancer cells. Further study suggested that GRB7 silencing could suppress gastric cancer tumor growth in vivo. Furthermore, our study uncovered an important interaction between GRB7 and MyD88. Silencing MyD88 was observed to alleviate the malignant phenotypes promoted by GRB7 in gastric cancer cells.

**Conclusions:**

Together, this study provided evidence that GRB7 may be an effective molecular targets for the treatment of gastric cancer.

**Supplementary Information:**

The online version contains supplementary material available at 10.1186/s12885-023-11694-5.

## Background

Gastric cancer is one of the most common malignancy, the third leading cause of deaths from cancer worldwide [1]. With the development of clinical diagnosis techniques, the detection and treatment rate of gastric cancer have been improved significantly [2]. However, traditional treatments including surgery, chemoradiotherapy and pharmacotherapy, cannot promise a complete cure due to tumor recurrence, the strong metastatic characteristic of gastric cancer tumor cells, injuring normal cells and being prone to drug resistance [3, 4]. Although immunotherapy, a novel treatment method, has shown promising results in gastric cancer treatment, there are substantial limitations and several challenges [5]. More regrettably, postoperative survival rate within 5 years for gastric cancer patients is less than 30% due to the recurrence and metastasis of gastric cancer [6]. Recent advancements in molecularly targeted therapies have yielded remarkable improvements in outcome for the gastric cancer treatment [7, 8], however, the mechanism of gastric cancer has not been fully elucidated and its prognosis is still poor. Thus, it is urgent to dissect the underlying mechanism in gastric cancer progression and detect new therapeutic targets for gastric cancer.

Growth factor receptor bound protein 7 (GRB7) is a modular Src homology domain 2 (SH2)-containing adaptor protein, which belongs to the Grb7 adaptor protein family [9, 10]. Moreover, GRB7 is composed of a proline rich N-terminal domain, a Ras-associating domain, a plekstrin homology (PH) domain, a C-terminal SH2 domain and a BPS domain [11]. It has been reported that GRB7 could interact with phospho-Tyr residues in different active tyrosine-kinase receptors and other phospho-proteins [9], and involved in the transduction of multiple signal paths during various physiological and pathological processes [10]. Previous findings have suggested that GRB7 was up-regulated in breast, blood, pancreatic, and esophageal cancer, and may contribute to the invasive potential of cancer cells [12]. Furthermore, GRB7 was identified as a driver for MEK inhibitor resistance in KRAS mutant colon cancer [13]. Recently, GRB7 has been studied as a therapeutic target in various cancers [11], which plays important roles in tumor development of cancers by regulating cell proliferation, apoptosis and cell migration [9]. Additionally, it also been reported that GRB7 is amplified in human gastric cancer [14]. However, the tumorigenic mechanism of GRB7 in the processes of gastric cancer is yet to be known.

Herein, we examined the expression levels of GRB7 in gastric cancer and para-normal tissues, and evaluated the correlation between GRB7 expression and clinicopathologic features, as well as analyzed prognosis of gastric cancer patients. Moreover, the pivotal roles of GRB7 in regulating gastric cancer cell viability, migration and apoptosis were investigated. We further performed in vivo experiments to verify our assumptions. In summary, our results suggest that GRB7 may be a valuable therapeutic target for gastric cancer.

## Materials and methods

### Immunohistochemical analysis

Gastric cancer tissues and para-normal tissues microarray chip (Shanghai Yibeirui Biomedical Science and Technology Co., Ltd) including 86 gastric cancer tissues and 117 para-carcinoma tissues was applied for IHC analysis. The study was approved by the Ethics Committee of The First Affiliated Hospital of Nanchang University. Written informed consents were obtained from all subjects before surgery. Briefly, the paraffin sections were baked at 65℃ for 30 min, dewaxed in xylene and hydrated with graded ethanol. Following antigen retrieval in the citrate buffer (pH 6.0) at 180℃ for 15 min, endogenous peroxidase was then blocked by 3% H_2_O_2_ for 5 min and goat serum for 15 min. Subsequently, sections were incubated with the primary antibody anti- GRB7 (1:100, Cat. SRP06265, Saierbio) or anti-Ki-67 antibody (1:200, Cat. # Ab16667, Abcam) at 4℃ overnight. In the next day, sections were washed 3 times for 5 min in 1xPBST, followed by incubation of secondary antibody (goat anti-rabbit IgG H&L (HRP), 1:400, Cat. # ab97080, Abcam) for 2 h at room temperature. The staining was visualized with diaminobenzidine in the dark, followed by a hematoxylin counterstain. Finally, stained tissues were viewed with ImageScope and CaseViewer at magnifications of 200× and 400× objective. Staining percentage ranks were categorized as: 1 (1–24%), 2 (25–49%), 3 (50–74%) and 4 (75–100%), which according to the scoring criteria described previously [15]. The expression parameters (high or low) were relative to the median expression at baseline.

### Cell lines

Human gastric cancer cell lines (AGS, MGC-803 and SGC-7901) and the normal gastric mucosal cell line (GES1) were purchased from the American type culture collection (ATCC) (https://www.atcc.org/). Cells were cultured in RPMI-1640 medium or DMEM (Gibco, USA) supplemented with 10% FBS (Invitrogen, USA). Meanwhile, all cell plates were incubated in a humidified incubator with 5% CO_2_ at 37℃.

### Short hairpin RNA (shRNA) transfection of cell lines AGS and MGC-803

Stable GRB7 knockdown AGS and MGC-803 cell models were generated by transfection of specific shRNA expression plasmids. Briefly, negative control (shCtrl) and shRNAs targeting GRB7 sequences (shCtrl: 5’-TTCTCCGAACGTGTCACGT-3’, shGRB7-1: 5’- TGTAGTAAAGGTGTACAGTGA-3’; shGRB7-2: 5’- TTGAGAAGTGCCTCAGATAAT-3’; shGRB7-3: 5’- CAGGACGGAAGCTTTGGAAAC-3’) were synthesized by Shanghai Bioscienceres Co., Ltd. (Shanghai, China). The shRNA sequences were inserted into BR-V-108 linearized vectors with green fluorescent protein (GFP) tag, and then transformed into *E. coli* TOP 10 competent cells (BioSCI Res). 293T cells were transfected with high purity plasmids for lentivirus production by using Lipofectamine 2000 reagent (Thermo Fisher Scientific) according to manufacturer’s instructions. Afterward, shGRB7 or the negative control (shCtrl) vectors were transfected into AGS and MGC-803 cells by using Lipofectamine® 3000 (Invitrogen, Carlsbad, CA, USA). After 48 h, transfection efficiency was evaluated using a fluorescent microscope and cells were harvested for subsequent research.

### Quantitative real time PCR (qRT-PCR)

The total RNA was abstracted from cells (gastric cancer cell lines AGS, MGC-803 and SGC-7901 and normal gastric mucosal cells GES1) using Trizol (Cat. # T9424-100m, Sigma) based on the manufacturer’s instruction. The quality of total RNA was quantified by Nanodrop 2000/2000 C (Thermo). Reverse-transcription was carried out using M-MLV kit (Promega Corporation, USA) and cDNA was then added into the reaction system for real-time PCR using SYBR green mastermixs kit (Vazyme) by applying 7500 sequence detection system (Applied Biosystems). The relative expression of GRB7 RNA was calculated by the 2^−ΔΔCt^ method, with GAPDH as an endogenous control. The primer sequences of NCAPG2 and GAPDH were displayed as following: GRB7-Forward: 5’-CTGGGGGTTCCTAGACGGAG-3’, GRB7-Reverse: 5’-AGAGAGGGGCTTAACGGAAC-3’; MYD88-Forward: 5’- TTACAGGTGGCCGCTGTAGA-3’, MYD88-Reverse: 5’- GGGGCAATAGCAGATGAAGG-3’; GAPDH-Forward: 5’- TGACTTCAACAGCGACACCCA-3’; GAPDH-Reverse: 5’- CACCCTGTTGCTGTAGCCAAA-3’.

### Western blotting

Lentivirus transfected AGS and MGC-803 cells were harvested and lysed in ice-cold IP lysis buffer, followed by determination of the protein concentration with a BCA Protein Assay kit (Cat. # 23,225, HyClone-Pierce). Subsequently, equal amount proteins (20 µg) were separated by 10% SDS-PAGE and transferred to PVDF membranes (Invitrogen). Subsequently, the membranes were meticulously transferred into their designated incubation containers, with each antibody specifically associated with a PVDF membrane. Prior to antibody hybridization, the membranes underwent cutting based on the molecular weight of the target protein, resulting in the absence of images of adequate length. After being blocked with TBST solution containing 5% skim milk for 45 min, PVDF membranes were incubated with primary antibodies at 4℃ overnight. The following day, membranes were further incubated with secondary antibodies for 1 h at room temperature. Finally, enhanced chemiluminescence (ECL, Amersham) was used to visualize the protein signal bands. GAPDH were selected as an endogenous reference. The primary antibodies were presented in table S2.

### Celigo cell count assay

Cell proliferation rate was assessed Celigo cell counting assay. In Brief, GRB7 was stably knocked down by shGRB7 in AGS and MGC-803 cell lines, and cells were plated into 96-well plates at the density of 2 × 10^3^ cells per well and cultured for 5 days. MEM medium was changed every 72 h. The GFP-positive cells were counted by using Celigo image cytometer (Nexcelom Bioscience) every day for five consecutive days once a day for 5 days and cell proliferation curve was graphed.

### Flow cytometry

Apoptosis were detected by flow cytometry assay. AGS and MGC-803 cells transfected with shGRB7 or shCtrl were cultured into 6-well plates for 3 days. Following incubation, cells were digested with trypsin, washed in ice-cold 1×binding buffer and then resuspended in 200 µL Hanks 1× binding buffer. Subsequently, 5 µL Annexin V-APC was added into 100 µL cell suspensions and incubated for 15 min in the dark. Finally, the apoptotic rate of cells was analyzed using a flow cytometer (BD Biosciences, USA).

### Human phospho-kinase array

To detect the relative phosphorylation levels of protein in AGS cell line with or without GRB7 knockdown, human phospho-kinase array kit (ARY003C, Bio-Techne, China) was performed according to manufactures’ instruction and as previously described [16]. In brief, AGS cells were lysed in lysis buffer and the membranes were blocked in array buffer for 1 h at room temperature. Subsequently, 2.0 mL diluted cell lysate were added into membranes and incubated overnight at 4 °C, followed by continue incubation with detection antibody cocktail A (DAC-A) or detection antibody cocktail B (DAC-B) for 2 h at room temperature. Afterwards, membranes were incubated with array buffer containing diluted streptavidin‑HRP for 30 min. Finally, each spot corresponding to the amount of phosphorylated protein bound was acquired using enhanced ECL (Amersham) and quantitated using Quantity One software (National Institute of Health).

### Wound healing assay

After transfection, AGS and MGC-803 cells in logarithmic phase were collected and digested with trypsin to prepare cell suspension. Subsequently, cells were cultured in 96-well plates at a density of 5 × 10^4^ cells/well. After 24 h, media was replaced by low concentration serum medium, 96 wounding replicator (Cat. VP408FH, VP scientific) was used to generate straight scratches gently in the lower central part of the 96-well plates. Following washing twice with PBS, the cells were maintained with serum-free medium. The wounded areas were observed under a microscope (200×), photographed at 0 h, 24 and 48 h time points after scratching. The migration distances were analyzed using a fluorescence-based Cellomics ArrayScan VTI analyzer (Thermo Fisher Scientific).

### Transwell assay

AGS and MGC-803 cells transfected with shGRB7 or shCtrl were seeded at a density of 5 × 10^4^ cells /ml. 100 µL serum-free medium were added in upper chamber of 24-well plates and placed in the incubator for 1 h at 37 ℃. The lower compartment of 24-well plates was filled with 600 µL medium containing 30% FBS. Subsequently, cells were re-suspended with serum-free medium and diluted to a certain concentration. After that, 100 µL resultant cell suspension (containing 1 × 10^5^~2 × 10^5^ cells) was added into each upper chamber and cultivated for 24 h. Finally, the fixed migrant cells were stained with Giemsa and photographed by microscopy.

### Xenograft animal model

BALB/c nude mice were provided by Beijing Vital River Laboratory Animal Technology Co., Ltd. The experimental protocols were approved by Ethics Committee of The First Affiliated Hospital of Nanchang University. Nude mice (four-week-old) were divided randomly into 2 groups (shCtrl or shGRB7 group) with 10 mice in each. Xenograft tumor models were established by subcutaneously injecting of 1 × 10^7^ stable MGC-803 cells transfected with shCtrl or shGRB7 into the left flank of nude mice. After tumor formation, the volume of the tumors were measured every three days during a total period of 42 days. Tumor volume was calculated as π/6×length×width2. Subsequently, mice were sacrificed by cervical dislocation and tumor tissues were extracted for weight measurement and IHC staining for GRB7 and Ki67.

### Co-expression analysis

The co-expressed genes of GRB7 in the human gastric cancer TCGA database were examined using cBioPortal (https://www.cbioportal.org). The RNA sequencing data were quantified by RSEM and were transformed by log2 (x + 1). Spearman correlation coefficient R (Spearman) between the levels of MyD88 and GRB7 was calculated using the cor.test function in the statistics package R, and the results were depicted with R package ggplot2.

### Co-IP assay

Cell were lysed by IP lysis buffer for 5 min on ice, and then centrifuged at 13,000 g. After determining the total protein concentration with BCA kit, antibodies were added to protein samples and incubated with rotation over night at 4 °C, followed by addition of beads for incubation for another 2 h. Subsequently, the protein complexes bound to antibodies were washed twice times with IP buffer at 4 °C, and then incubated with IP buffer and 5xloading buffer at 100 °C for 5 min. Finally, proteins eluted from the beads were subjected to Western blot analysis. The antibodies were presented in table S3.

### Statistical analyses

Statistical analysis was performed by GraphPad 6.0 software (GraphPad Prism, San Diego, CA, USA). Data were shown as mean ± SD, and differences between groups were analyzed by Student’s t-test. P < 0.05 was considered to indicate a statistically significant difference. Mann–Whitney U analysis and Spearman rank correlation analysis were used to assess the correlation between GRB7 expression and tumor characteristics in gastric cancer patients. Survival curves were obtained by the Kaplan–Meier method. All cell experiments were performed in triplicate.

## Results

### GRB7 is up-regulated in gastric cancer tissues and cells

**Fig. 1:**
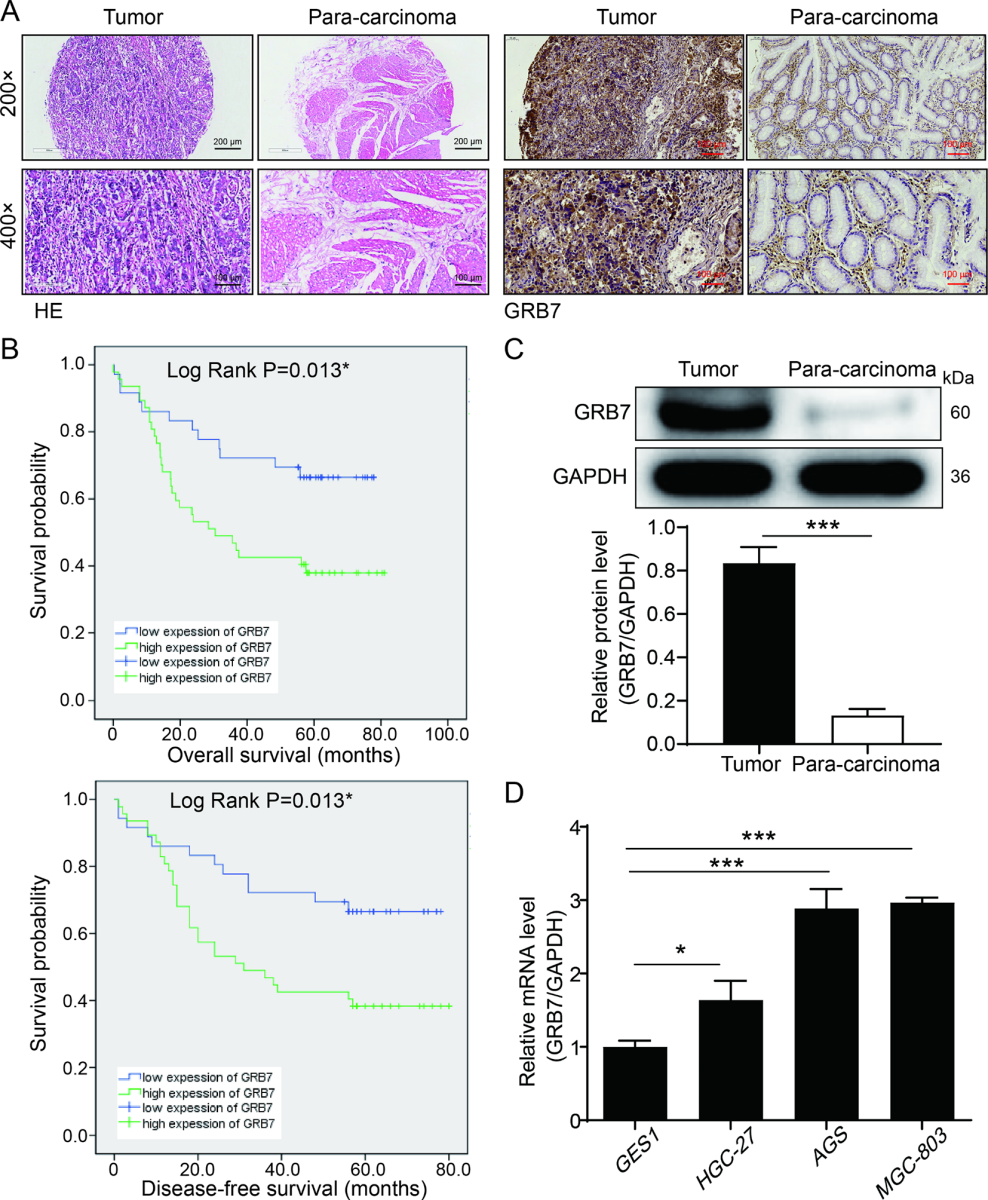
GRB7 is up-regulated in gastric cancer tissues and cells. (**A**) Representative images of HE and IHC staining of human gastric cancer tumor tissues and para-carcinoma tissues. (**B**) The expression levels of GRB7 in gastric cancer markedly impacted the survival time of patients (OS: overall survival; DFS, disease-free survival). (**C**) The protein expression levels of GRB7 in tumor tissues and corresponding normal tissues was assessed through western blot analysis. (**D**) The background expression of GRB7 in gastric cancer cell lines and normal gastric mucosal cells was detected by qPCR. * P < 0.05, *** P < 0.001. Error bars are the SD for three technical replicates

The pathological status of cancerous and normal tissues were initially evaluated by HE staining. As shown in Fig. 1A, gastric cancer tissues exhibited irregular nuclear morphologies, deep chromatin staining, and heterogeneous cytoplasm, while para-normal tissues displayed normal nuclear morphologies, uniform staining, and orderly cell arrangement. IHC staining were then employed to detect the expression pattern of GRB7 in gastric cancer tissues and para-carcinoma tissues. It was observed that tumor cells showed strong expression of GRB7, while para-carcinoma tissue cells presented low IHC staining intensity (Fig. 1A). More importantly, GRB7 was highly expressed in the subtypes of gastric cancer in comparison to the para-carcinoma tissues (Table 1 and Table S1). Moreover, Mann-Whitney U analysis and Spearman correlation analysis revealed the correlation of GRB7 expression with age and T Infiltrate (Tables 2 and 3). Kaplan-Meier analyses revealed that the high GRB7 expression was significantly relates to a poor survival of gastric cancer patients (Fig. 1B). Subsequently, the upregulation of GRB7 in gastric cancer tissue was confirmed at the protein level through WB analysis (Fig. 1C). The mRNA levels of GRB7 expression was also higher in gastric cancer cell lines AGS, MGC-803 and SGC-7901 compared to GES1 cells (Fig. 1D). Taken together, our results showed that GRB7 is increased in gastric cancer and may have an important role in its development.

**Table 1:** Expression patterns in gastric cancer tissues and para-carcinoma tissues revealed in immunohistochemistry analysis

ERCC6 expression	Tumor tissue		Para-carcinoma tissue		p value
	Cases	Percentage	Cases	Percentage	
Low	38	44.2%	109	93.2%	
High	48	55.8%	8	6.8%	< 0.001

**Table 2:** Relationship between GRB7 expression and tumor characteristics in patients with gastric cancer

Features	No. of patients	GRB7 expression		p value
		low	high	
All patients	86	38	48	
Age (years)				0.006
≤ 64	40	24	16	
> 64	46	14	32	
Gender				0.772
Male	58	25	33	
Female	28	13	15	
T Infiltrate				0.012
T1	5	4	1	
T2	11	5	6	
T3	49	25	24	
T4	21	4	17	
Lymphatic metastasis (N)				0.814
N0	16	6	10	
N1	15	10	5	
N2	16	5	11	
N3	39	17	22	
Stage				0.163
I	7	4	3	
II	25	13	12	
III	53	21	32	
IV	1	0	1	
Number of lymph node metastases				0.360
≤ 6	45	22	23	
> 6	41	16	25	

**Table 3:** Relationship between GRB7 expression and tumor characteristics in patients with gastric cancer

		GRB7
T Infiltrate	Spearman correlation	0.297**
N	Significance (two-tailed)	0.005
N	N	86
	Spearman correlation	0.271*
	Significance (two-tailed)	0.012
	N	86

### Knockdown efficacy of GRB7 in AGS and MGC-803 cells

**Fig. 2:**
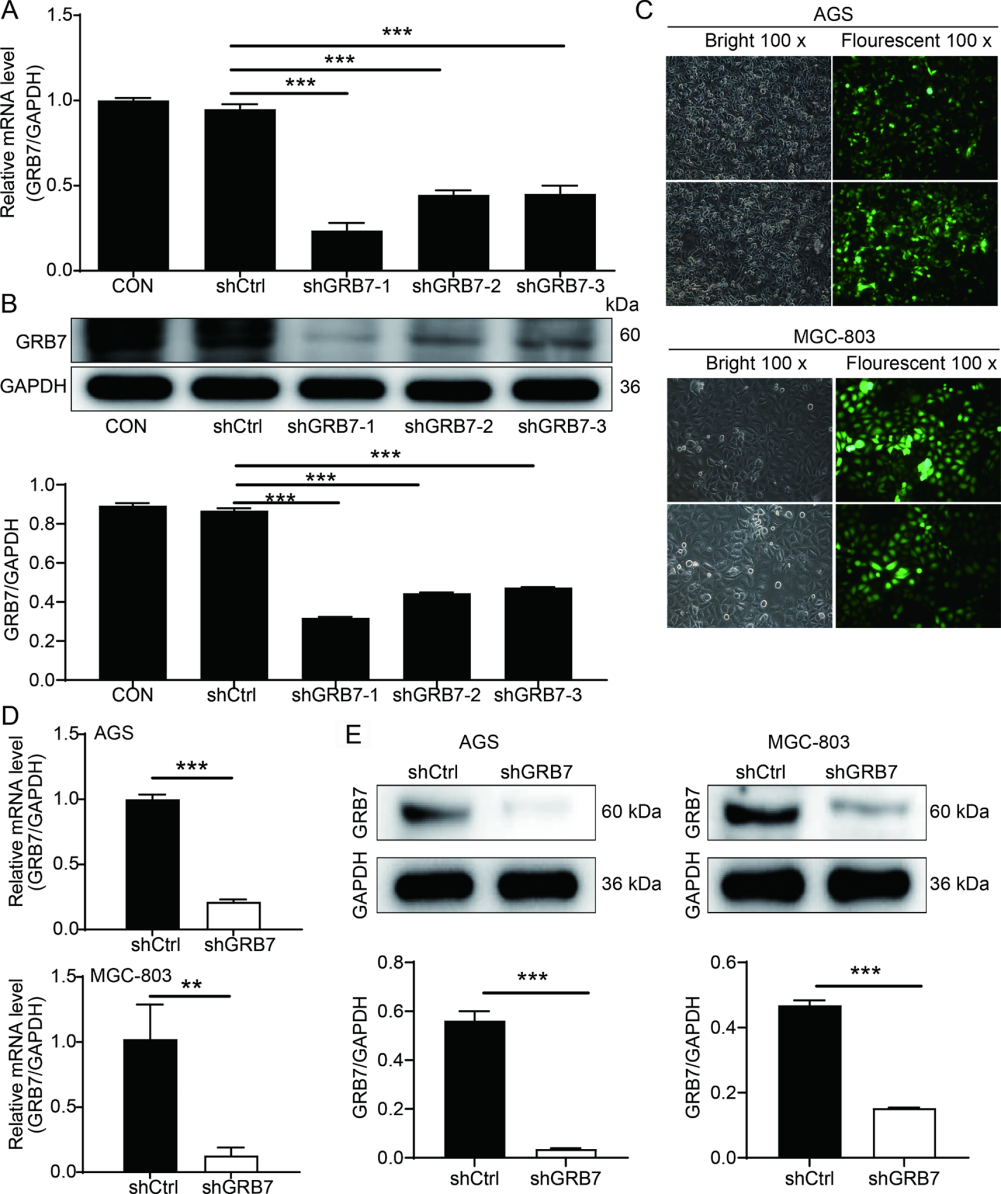
Knockdown Efficacy of GRB7 in AGS and MGC-803 cells. (**A**-**B**) The efficiency of 3 shRNAs targeting GRB7 was evaluated by qPCR (**A**) and western blot (**B**). (**C**) GFP expression levels were observed 72 h following shGRB7 and shCtrl LV transfection in AGS and MGC-803 cells. (**D** and **E**) The knockdown efficiency of GRB7 in AGS and MGC-803 cells was evaluated by qPCR (**D**) and further verified by western blot (**E**). Results were presented as mean ± SD. Error bars are the SD for three technical replicates. **P < 0.01, ***P < 0.001

To investigate the role of GRB7 in gastric cancer progression, cell models for GRB7 knockdown were constructed through the transfection of shCtrl or shGRB7 in AGS and MGC-803 cells. Briefly, the knockdown efficiency of three shRNAs targeting GRB7 were determined by qPCR in AGS cells. As shown in Fig. 2A-B, shGRB7-1 showed the highest knockdown efficiency, among the three shRNAs, and was thus selected for subsequent experiments. Fluorescence imaging confirmed successful transfection with GRB7 in both AGS and MGC-803 cell lines. (Fig. 2C). Next, qPCR and western blotting assays were conducted to evaluate the knockdown efficiency on the mRNA and protein levels of GRB7, respectively. The GRB7 mRNA level in shGRB7 group was significantly decreased by 78.69% compared the shCtrl group in AGS cells (P < 0.001), and a strong reduction by 87.50% in MGC-803 cells (P < 0.001) (Fig. 2D). Similarly, the protein levels of GRB7 in both cell lines transfected with shGRB7 were reduced as compared with control cells (Fig. 2E). Together, these results demonstrated the successful establishment of GRB7 knockdown model, which could be used for the following functional studies.

### GRB7 knockdown inhibited gastric cancer development in vitro

**Fig. 3:**
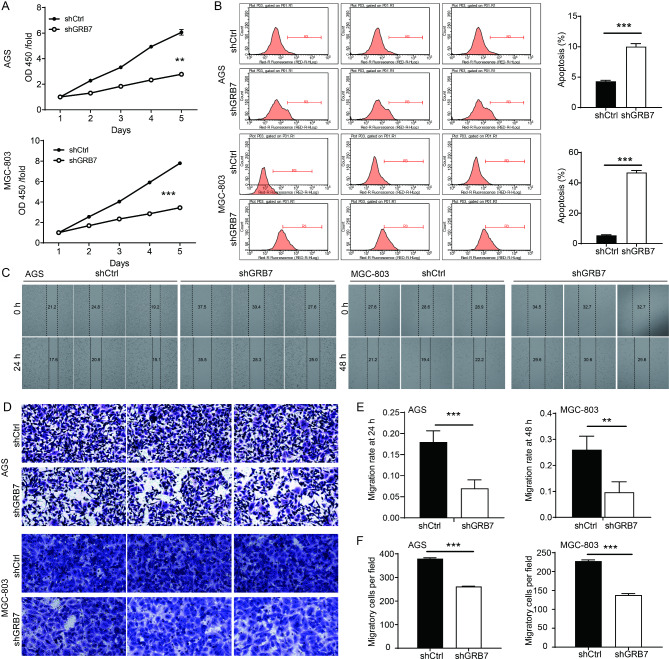
GRB7 silencing inhibited gastric cancer development in vitro. (**A**) The effects of GRB7 knockdown on cell proliferation of AGS and MGC-803 cells were evaluated by Celigo cell counting assay. (**B**) The flow cytometry was utilized to analyze cell apoptosis upon GRB7 knockdown. Results were presented as mean ± SD. Error bars are the SD for three technical replicates. *P < 0.05, **P < 0.01, ***P < 0.001. (**C**-**D**) Wound-healing (**C**) and Transwell assays (**D**) were utilized to assess the effects of GRB7 knockdown on cell migration of AGS and MGC-803 cells. Representative pictures were taken for each group at 200×magnification (**D**). (**E**-**F**) Quantified results of wound-healing (**E**) and Transwell assays (**F**). Results were presented as mean ± SD. Error bars are the SD for three technical replicates. ***P < 0.001

To investigate the effect of GRB7 knockdown on the cell proliferation in AGS and MGC-803 cell lines, a Celigo analysis was performed. At 5 days after transfection, the proliferation of both cell lines that were transfected with shGRB7 was significantly reduced compared with cells transfected with shCtrl (P < 0.001) (Fig. 3A). The role of GRB7 in regulating cell apoptosis was then investigated using flow cytometry. As revealed in Fig. 3B, compared with the shCtrl groups, GRB7 silencing resulted in an increase the proportion of apoptotic cells in both cells (fold change = 2.3 and 8.6 in AGS and MGC-803, respectively, P < 0.001 for both). The effects of GRB7 on cell migration of AGS and MGC-803 cells was further investigated by wound healing assay (Fig. 3C) and Transwell assays (Fig. 3D). As revealed in Fig. 3E, downregulation of GRB7 significantly reduced the wound closure rates in the wound-healing assay. Transwell assays showed similar results. In comparison to the shCtrl group, GRB7 knockdown significantly reduced the migration ability by 31% in AGS cells and 39% in MGC-803 cells, respectively (Fig. 3F). Collectively, these results indicated that the GRB7 knockdown inhibited the progression of gastric cancer.

### GRB7 knockdown suppressed gastric cancer tumorigenesis

**Fig. 4:**
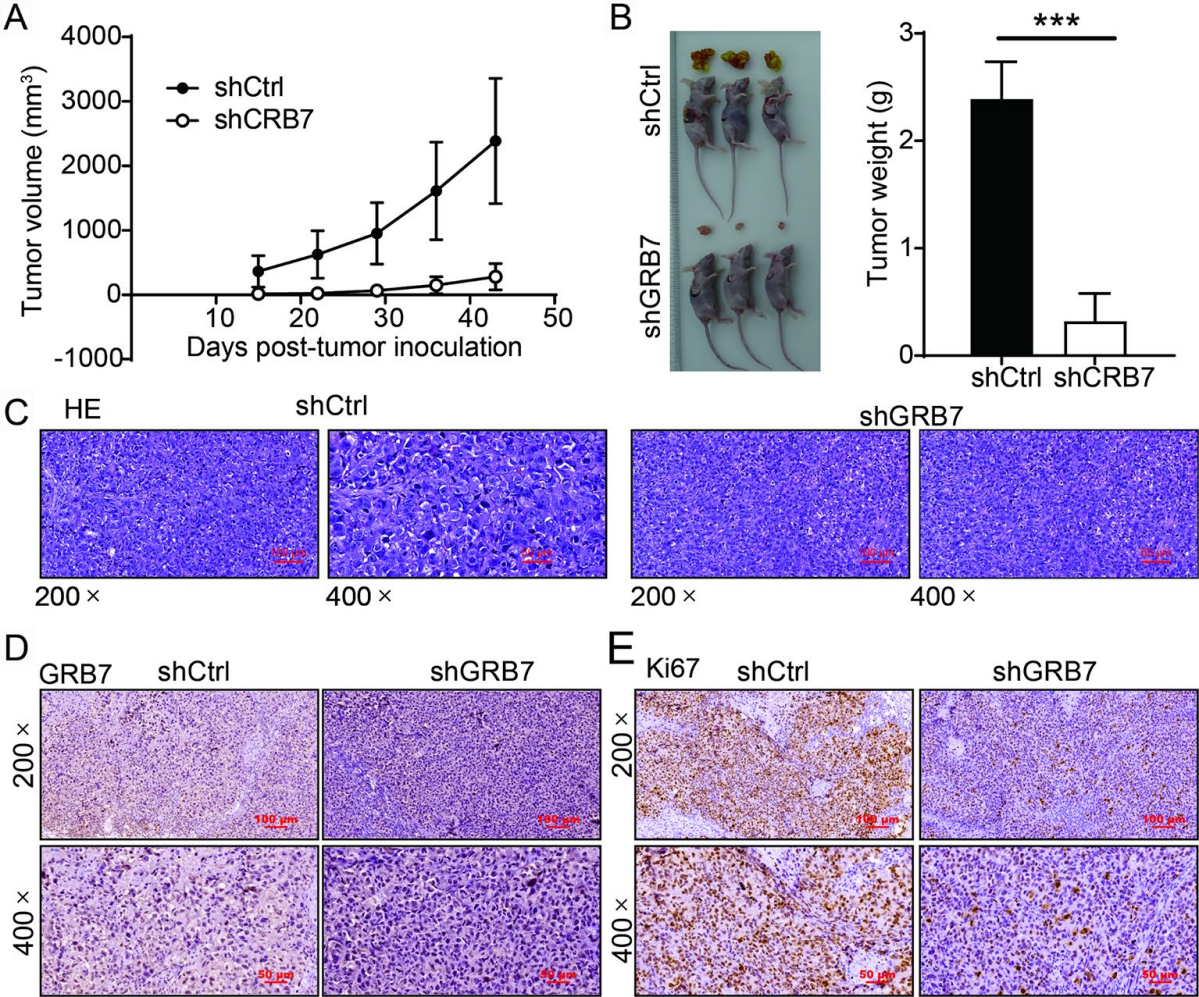
GRB7 silencing suppressed gastric cancer tumorigenesis. (**A**) The tumor volumes were measured throughout culture of animal models. (**B**) The photos of tumors were taken after the removal of tumors. Tumor weights were measured after sacrificing the mice models. (**C**) The results of HE staining of tumor tissues of nude mice. (**D**-**E**) GRB7expression (**D**) and Ki67 expression (**E**) was detected by IHC in shCtrl and shGRB7 group. Results were presented as mean ± SD. Error bars are the SD for three technical replicates. ***P < 0.001

To further investigate the effects of GRB7 on gastric cancer tumorigenesis, we constructed a subcutaneous tumorigenesis model in vivo following GRB7 knockdown. Briefly, MGC-803 cells transfected with shGRB7 or shCtrl vectors were injected into nude mice. The tumor volume and body weights were measured every 3 days. As shown in Fig. 4A, silencing of GRB7 significantly induced slower growth of tumors and reduced the tumor volumes compared to those in the shCtrl group. After 6 weeks, the mice were sacrificed and the tumors were weighed. We observed that the average weight of shGRB7 group mice was significantly decreased compared to the shCtrl group (Fig. 4B). In the shCtrl group, the results of HE staining revealed the presence of a greater number of necrotic areas, characterized by the breakdown of cell bodies. Conversely, the tissue from mice in the shGRB7 group exhibited well-organized cells and typical cellular structure. (Fig. 4C). Next, we examined the expression of GRB7 in mouse tissues by IHC staining, and found that down-regulation of GRB7 expression levels in shGRB7 group mice (Fig. 4D). Finally, we analyzed the proliferative activity of the tumor cells by incubating the slides with an anti-Ki-67 antibody. As shown in Fig. 4E, the expression of Ki67 was lower in the shGRB7 group compared to the shCtrl group. Altogether, these findings indicated that GRB7 knockdown significantly suppressed gastric cancer tumor growth.

### GRB7 regulated GC progression via MyD88

**Fig. 5:**
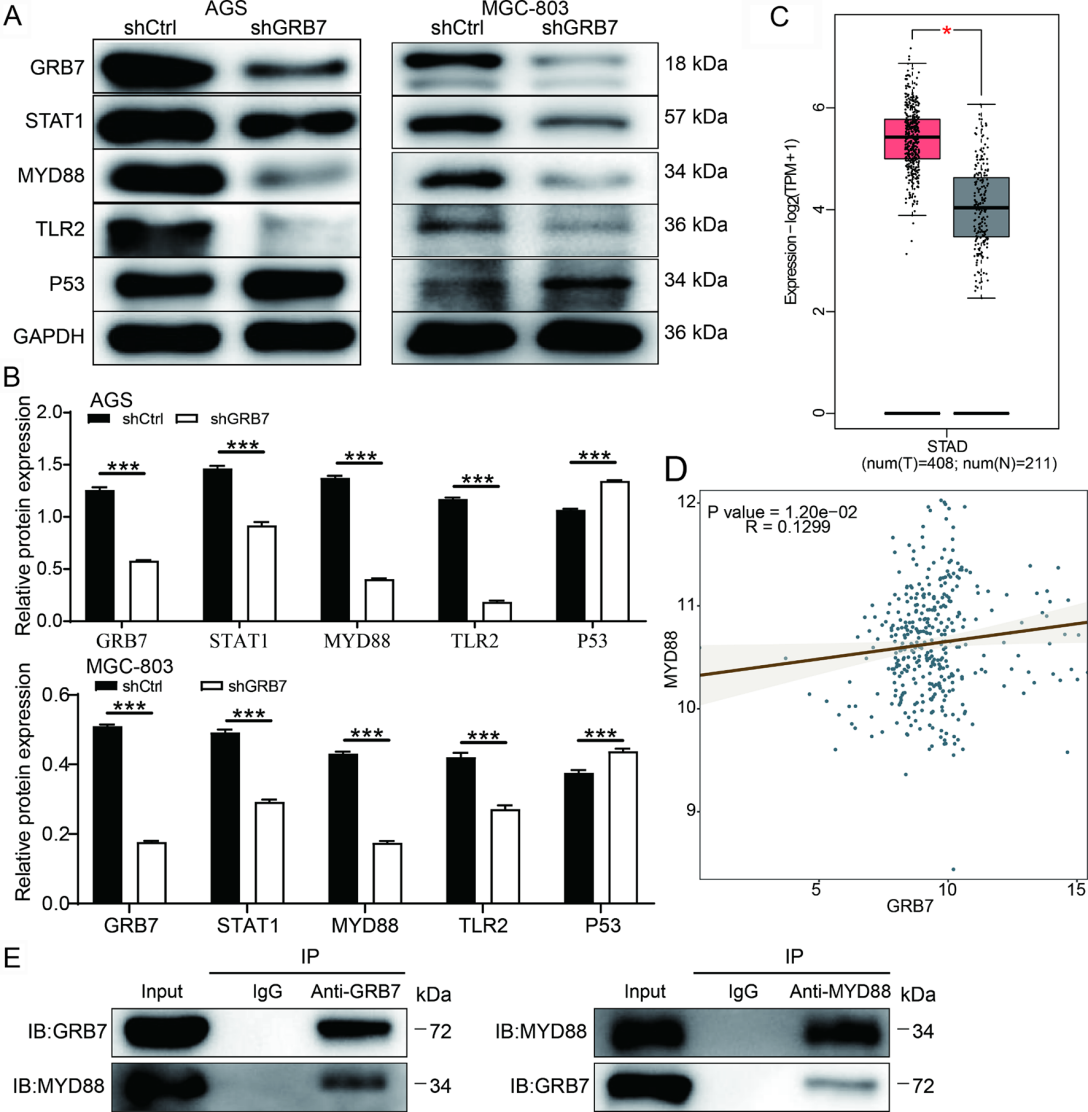
GRB7 positively regulated the expression of MYD88 in gastric cancer. (**A**) The expression of key proteins of TLR family signaling pathway was detected by western blot. GAPDH was used as the loading control. (**B**) Grayscale analysis of the protein bands. (**C**) The expression level of MYD88 was analyzed using the RNA-seq data downloaded from TCGA. (**D**) Correlation analyses between the expression of GRB7 and MYD88. (**E**) The binding of GRB7 by MYD88 was verified in AGS sells via a coimmunoprecipitation (Co-IP) assay. Results were presented as mean ± SD. Error bars are the SD for three technical replicates. * P < 0.05, ** P < 0.01, ***P < 0.001

To further identify the potential downstream functional proteins of GRB7, we subsequently performed the Human Phospho-Kinase Array assay in MGC-803 cells with GRB7 knockdown. It was found that GRB7 knockdown downregulated the phosphorylation levels of proteins within the TLR signaling pathway, including MyD88, STAT1, STAT2, TLR2 proteins (Fig. S1A). Consistently, WB results showed that GRB7 knockdown induced up-regulation of p53, and down-regulation of STAT1, MyD88, TLR2, especially MyD88 (Fig. 5A-B). Notably, MyD88 is an intracellular adaptor protein that highly expressed in gastric cancer (Fig. 5C), and played a critical role in gastric cancer progression [17], we thus focused on the MyD88 in the following analysis. The TCGA data revealed that MyD88 expression was positively correlated with the GRB7 expression in gastric cancer tumor samples (Fig. 5D). Subsequently, the interaction between MyD88 and GRB7 was confirmed in AGS cells using a Co-IP assay (Fig. 5E). Additionally, GRB7 knockdown significantly reduced the expression of MyD88 at mRNA level (Fig. S1B). Through single-gene GSEA enrichment analysis, the inflammation and apoptosis pathways were significantly enriched within the GRB7 and MyD88 gene set (Fig. S2). Therein, we speculated that GRB7 may affect MyD88 expression to regulate gastric cancer progression.

**Fig. 6:**
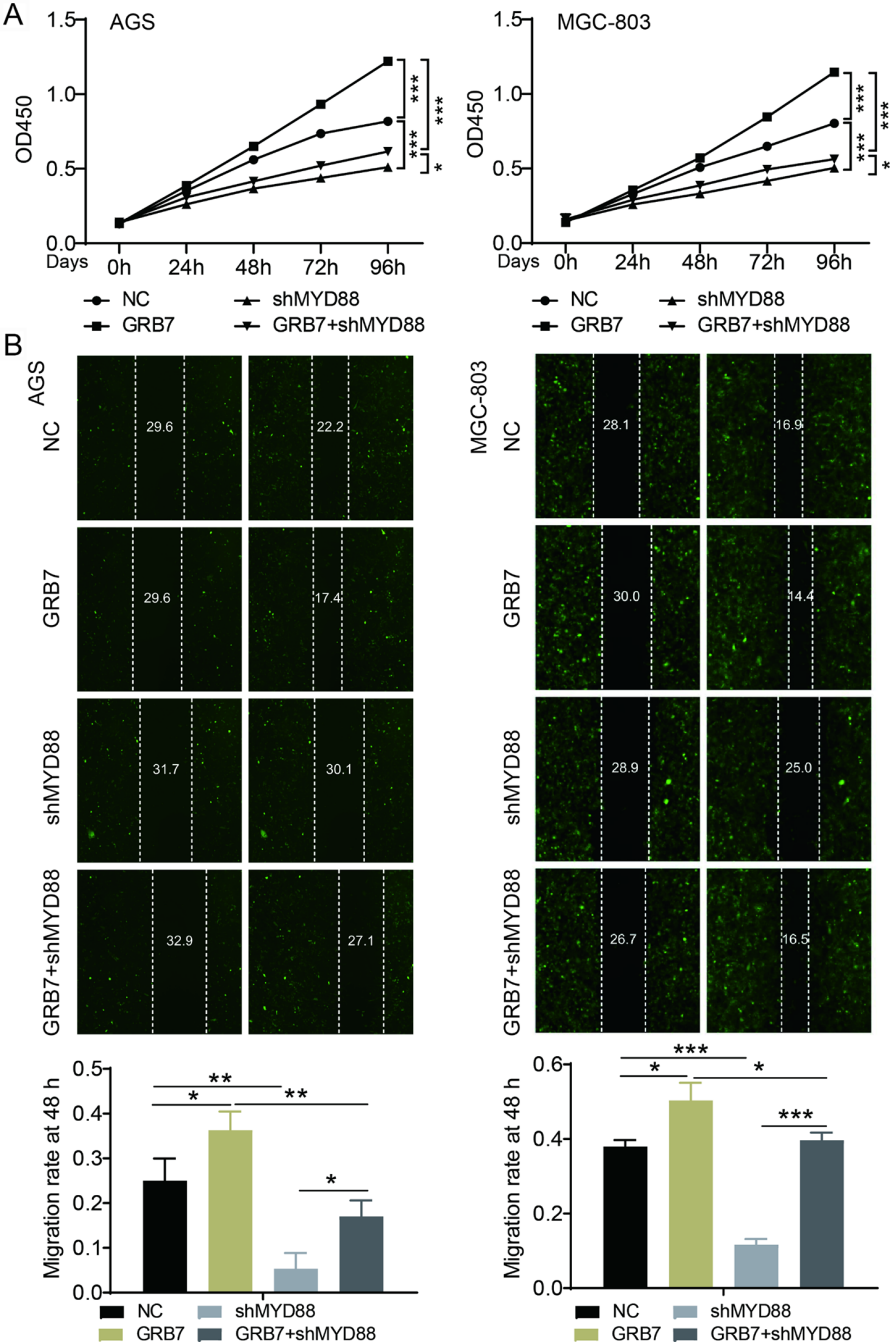
GRB7 facilitated gastric cancer cell proliferation and migration through regulation of MyD88. The functional experiments were conducted in the four groups of hepatocyte-like cells, including control, GRB7 overexpression, MyD88 knockdown and GRB7 overexpression with MyD88 knockdown. (**A**) Proliferation by CCK8 assay. (**B**) Cell migration detected by wound healing assay. Results were presented as mean ± SD. Error bars are the SD for three technical replicates. * P < 0.05, ** P < 0.01, ***P < 0.001

To verify this, we performed functional experiments with MyD88 knockdown after GRB7 overexpression. We demonstrated that GRB7 overexpressing significantly enhanced cell proliferation and migration in AGS and MGC-803 cells (Fig. 6A-B).

In contrast, MyD88 knockdown repressed cell proliferation and migration in two cell lines (Fig. 6A-B). Intriguingly, MyD88 depletion significantly weakened the promotive effect of GRB7 overexpression on malignant phenotypes of gastric cancer cells (Fig. 6A-B). Together, these results suggest MyD88 may contribute to GRB7-mediated gastric cancer progression.

## Discussion

In this study, we aimed to investigate the potential role of GRB7 in the regulation of gastric cancer progression. It was found that GRB7 expression was up-regulated in gastric cancer and negatively correlated with overall survival in these patients. The results of functional experiments further revealed that GRB7 knockdown significantly inhibited the proliferative and migratory abilities of gastric cancer cells in vitro, as well as suppressed the tumor growth of gastric cancer in vivo. Collectively, these results uncovered that GRB7 may become an effective therapeutic target for the treatment of gastric cancer.

GRB7 as a functional multidomain adaptor protein, which interacts with phospho-tyrosine-related signaling molecules [18]. Aberrant expression of GRB7 has been investigated in a variety of cancers, such as oesophageal adenocarcinoma [19], thyroid cancer [20], colorectal cancer [21]. In our study, GRB7 was significantly highly expressed in gastric cancer, and the prognosis of patients with high GRB7 expression was much worse than that of patients with low GRB7 expression. Thus, we speculated that GRB7 may be used as a predictor prognosis of gastric cancer. Nevertheless, the sample size of this research is relatively small and should be expanded to verify our conclusion. It has been reported that GRB7, an important targetable factor, participates in varied physiological and pathological processes in human cancers [22]. Moreover, PH domain of GRB7 has protein and/or lipid binding abilities and regulate various cellular functions [18]. It has been also reported that GRB7 plays an important role in cell survival and growth [23, 24]. Based on this, the function roles of GRB7 in gastric cancer was investigated by a series of in vitro and in vivo experiments following GRB7 knockdown.

Previous study has shown that high GRB7 expression significantly promoted proliferation and tumorigenesis of bladder cancer [10]. In oesophageal adenocarcinoma, it has been demonstrated that reducing GRB7 leads to a decrease in proliferation and clonogenic survival, as well as the induction of cell apoptosis [19]. As mentioned above, our study discovered that GRB7 knockdown resulted in reduced cell proliferation and increased cell apoptosis in gastric cancer. It is worth noting that p53 has been shown to regulate cell cycle arrest, cellular senescence or apoptosis, and is considered to an activator of cell apoptosis [25]. Our study revealed that GRB7 knockdown significantly elevated the protein levels of p53 protein. Ki67, a cellular marker that indicates higher proliferation rates, was also found to be highly expressed in gastric cancer [26]. Our in vivo results supported the idea that depletion of GRB7 suppressed tumor growth and downregulated Ki67 expression in gastric cancer tumor tissue of mice. Additionally, Wang et al. found that GRB7 facilitated the migration and invasion of ovarian cancer [27]. Similarly, the results of this study also indicated that knocking down GRB7 reduced the ability of cell migration in gastric cancer cells. Based on these aforementioned findings, it is clear that GRB7 have a significant role in gastric cancer progression.

MyD88, which is a critical adaptor protein in the TLR family signaling pathway, plays a significant role in controlling immune responses and inflammation [28]. It holds great therapeutic value in the development of anti-cancer drugs [29]. Moreover, the activation of the NF-κB pathway by MyD88 has been found to contribute to the progression of gastric cardia carcinogenesis [30]. Additionally, a genetic defect in MyD88 has been identified in gastric cancer and may increase the risk of developing this type of cancer [31]. In this study, there was a significant positive correlation between the expression of MyD88 and GRB7. When the expression of GRB7 was downregulated, it led to a decrease in MyD88 expression. MyD88 is important for tumor cell growth and metastasis [32]. Our in vitro functional assays further revealed that MyD88 partially reversed the promotion of cell proliferation and migration caused by GRB7 overexpression in gastric cancer. These results preliminarily showed that GRB7 may regulate the progression of gastric cancer by specifically targeting MyD88.

In this study, GRB7 was observed to be frequently up-regulated in gastric cancer cells and tissues, and high GRB7 expression was associated with T Infiltrate and poor prognosis. When GRB7 was suppressed, it weakened the abilities of proliferation and migration in gastric cancer cells, increased the levels of apoptosis, as well as inhibited the tumor growth in vivo. Furthermore, it was discovered that GRB7 regulated malignant cellular characteristics through MyD88 in gastric cancer. In conclusion, the deletion of GRB7 hindered the progression of gastric cancer, indicating that it may be a promising candidate target with potential therapeutic value.

### Electronic supplementary material

Below is the link to the electronic supplementary material.

Supplementary Material 1: Supplementary Tables and Figures

Supplementary Material 2: Western blot raw images

## Data Availability

The data set supporting the results of this article are included within the article.

## List of abbreviations

GRB7: Growth factor receptor bound protein 7
IHC: Immunohistochemical
TMA: Tissue microarray
qRT-PCR: Quantitative reverse transcription PCR
MyD88: Myeloid differentiation primary response 88
STAT1: Signal transducer and activator of transcription 1
STAT2: Signal transducer and activator of transcription 2
TLR2: Toll-like receptor 2
p53: Tumor protein p53
Ki-67: Marker of proliferation Ki-67
Co-IP: co-immunoprecipitation
