# Supplementary material for: GRB7 plays a promoting role in the progression of gastric cancer
Source: BMC Cancer. 2023 Dec 21;23:1262. doi: 10.1186/s12885-023-11694-5 (PMC10734061; doi:10.1186/s12885-023-11694-5)
Supplement: Supplementary file 1 — Supplementary Material 1: Supplementary Tables and Figures [file 12885_2023_11694_MOESM1_ESM.pdf]

Supplementary materials

Table S1 The expression of GRB7 subtypes of the gastric cancer tissues

Pathological Subtype	Total Cases	High Expression (Score)	Low Expression (Score)	P-value
Poorly differentiated adenocarcinoma	20	13 (5.2)	7 (2.0)	<0.001
High-intermediate differentiated adenocarcinoma	3	2 (5.5)	1 (2.0)	
Low adhesive carcinoma (signet ring cell carcinoma)	16	10 (4.9)	6 (1.8)	<0.001
Intermediate-poorly differentiated adenocarcinoma	22	12 (6.7)	10 (2.1)	<0.001
Moderately differentiated adenocarcinoma	25	11 (6.0)	14(1.9)	<0.001

Table S2 Antibodies applied in Western blot of this research.

Primary antibody	Size/kDa	Diluted multiples	Source	Company	Catalog No.
GRB7	60	1:1000	Rabbit	Saier biolabas	SRP06265
STAT1	87	1:2000	Rabbit	Abcam	ab109320
MYD88	33	1:1000	Rabbit	Abcam	ab133739
TLR2	84	1:500	Rabbit	Bioss	bs-1019R
P53	53	1:500	Rabbit	Bioss	bs-8687R
GAPDH	36	1:30000	Mouse	Proteintech	60004-1-lg
Secondary		Diluted		Company	Catalog No.

antibody	multiples		
Goat Anti-Rabbit	1:3000	Beyotime	A0208
Goat Anti-Mouse	1:3000	Beyotime	A0216

Table S3 Antibodies applied in Co-IP assay of this research.

Primary antibody	Size/kDa	Diluted multiples	Source	Company	Catalog No.
Ubiquitin		1:1000	Mouse	CST	3936S
C-JUN		1:50	Rabbit	Abcam	ab40766
JUN	36	1:1000	Rabbit	Proteintech	24909-1-AP
GAPDH	36	1:30000	Mouse	Proteintech	60004-1-lg
RRP9	52	1:50/1:3000	Rabbit	invitrogen	A303-970A
MDM2	75-85	1:3000	Rabbit	Proteintech	27883-1-AP
GAPDH	36	1:30000	Mouse	Proteintech	60004-1-lg
Secondary antibody		Diluted multiples		Company	Catalog No.
Goat Anti-Rabbit		1:3000		Beyotime	A0208
Goat Anti-Mouse		1:3000		Beyotime	A0216

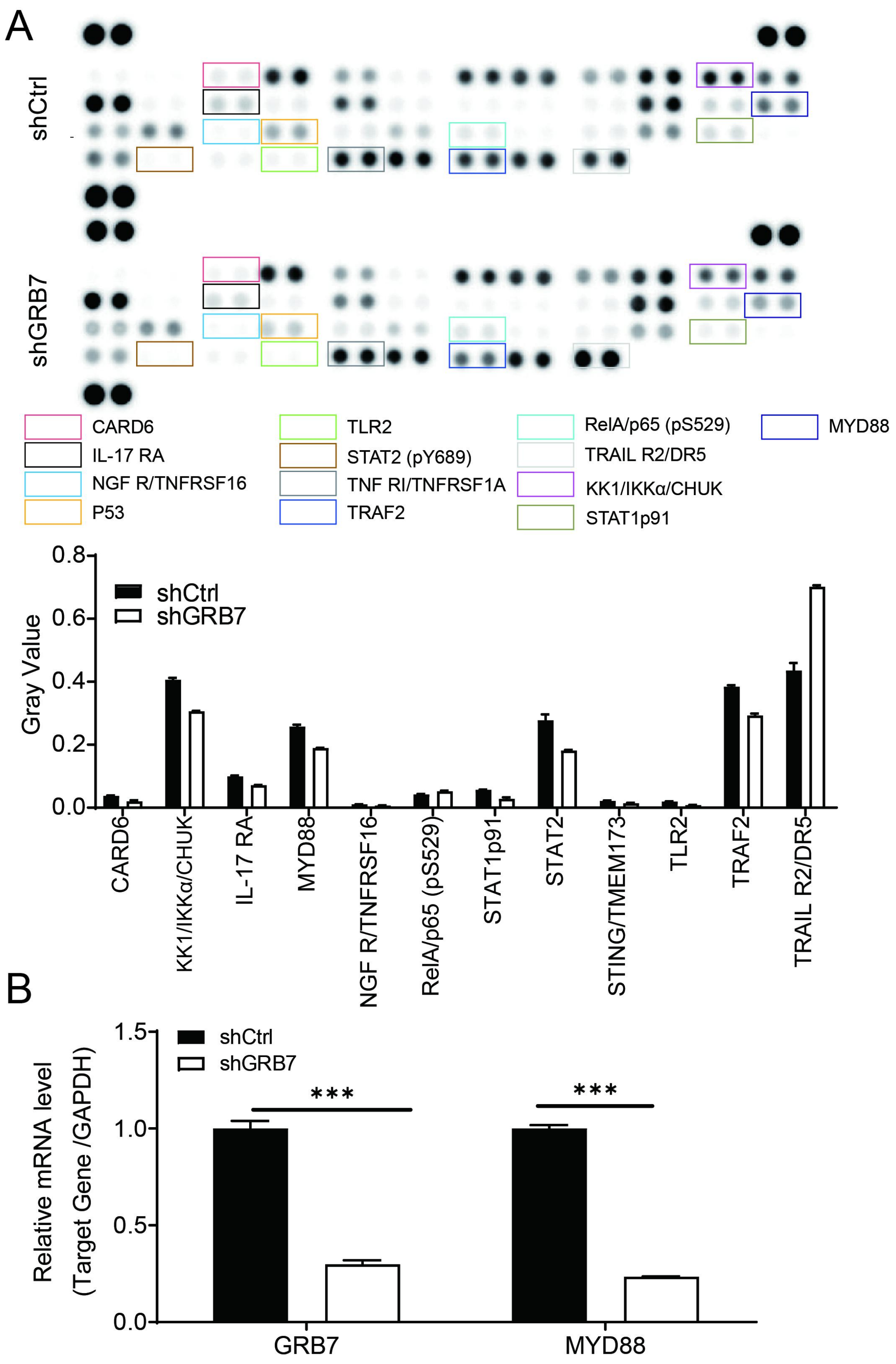

Fig. S1 GRB7 knockdown reduced the expression of MYD88

(A) The relative phosphorylation levels of related proteins in MGC-803 cells with or without GRB7 knockdown were analyzed by the human phospho-kinase array. (B)

Knockdown of GRB7 downregulated MyD88 expression at mRNA levels. Results were presented as mean ± SD. Error bars are the SD for three technical replicates. ***P < 0.001.

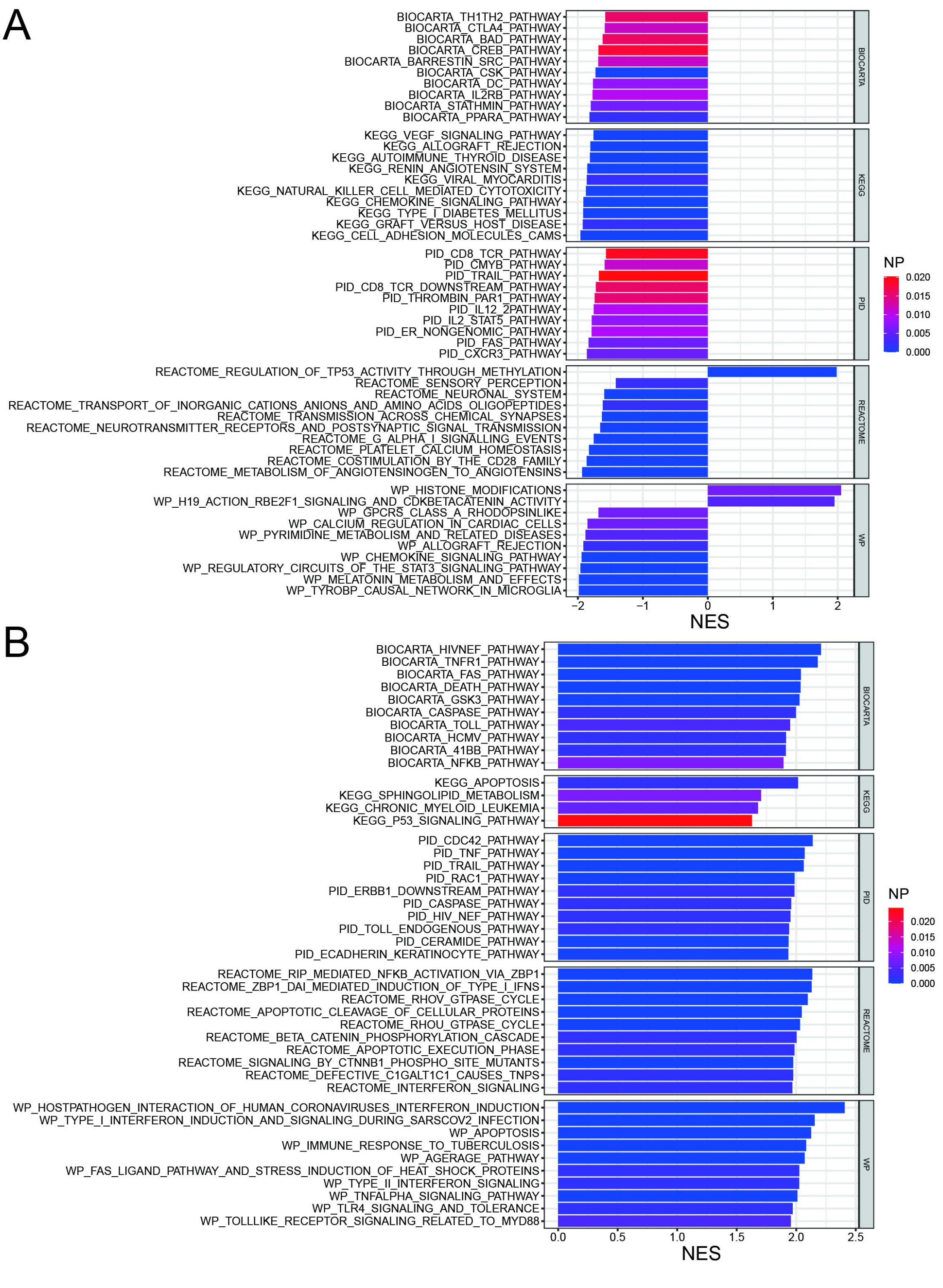

Fig. S2 Gene set enrichment analysis (GSEA) based on the KEGG gene set

(A) Significantly differential pathways associated with GRB7 in single-gene GSEA

enrichmen t analysis. (B) Through single-gene GSEA enrichment analysis, the involved signaling pathways included the inflammation and apoptosis pathways were significantly enriched within the MyD88 gene set.
