# Supplementary material for: GRB7 plays a promoting role in the progression of gastric cancer
Source: BMC Cancer. 2023 Dec 21;23:1262. doi: 10.1186/s12885-023-11694-5 (PMC10734061; doi:10.1186/s12885-023-11694-5)
Supplement: Supplementary file 2 — Supplementary Material 2: Western blot raw images [file 12885_2023_11694_MOESM2_ESM.pdf]

Figure 1C

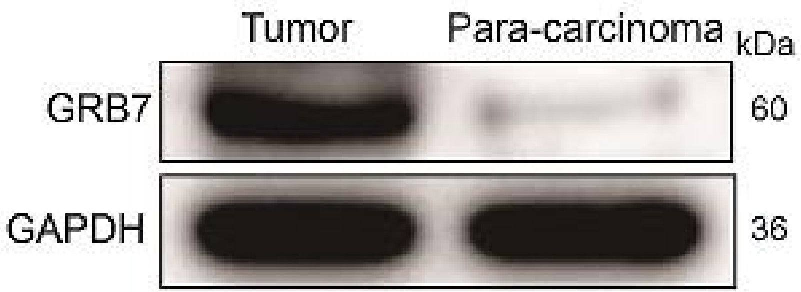
Tumor
Para-carcinoma
kDa
GRB7
60
GAPDH
36

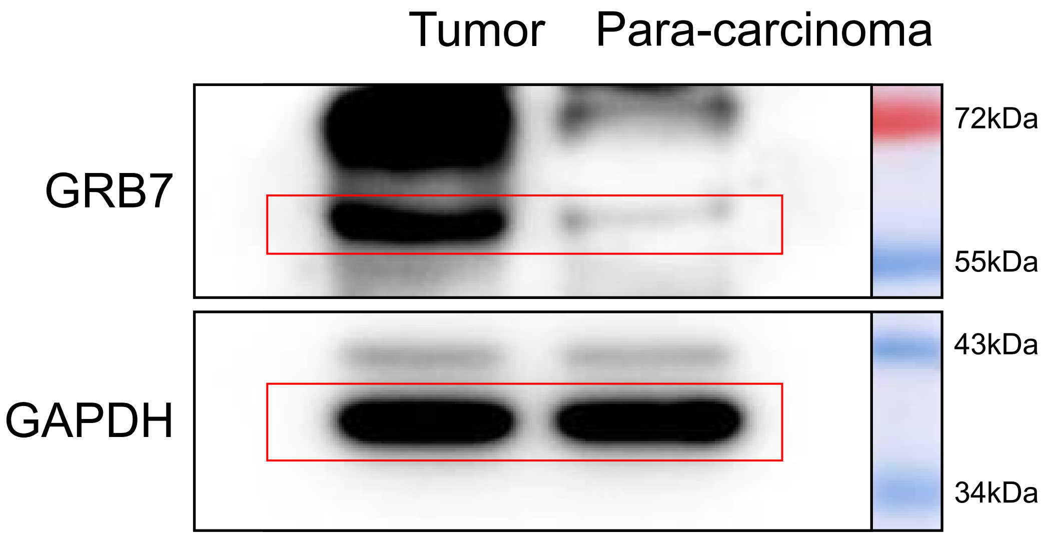
Tumor
Para-carcinoma
GRB7
72kDa
55kDa
GAPDH
43kDa
34kDa

Figure 2B

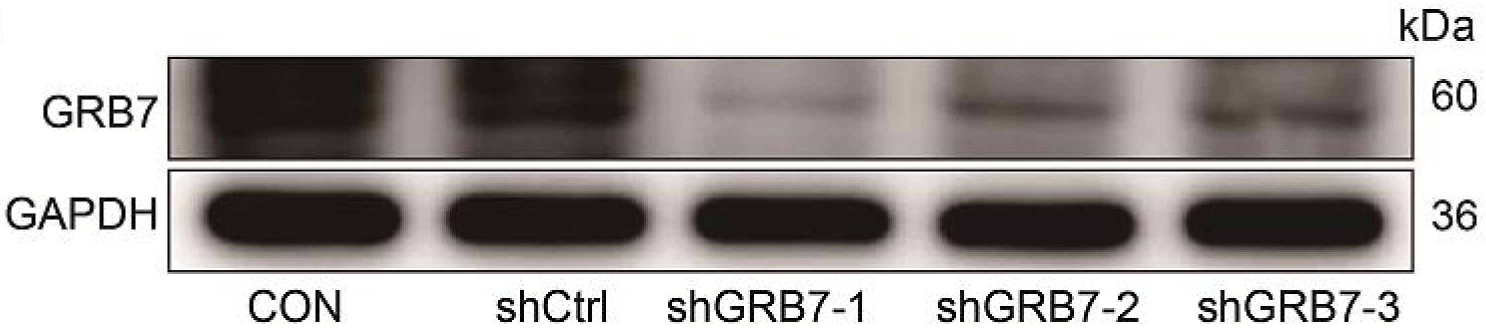

kDa
GRB7
60
GAPDH
36
CON
shCtrl
shGRB7-1
shGRB7-2
shGRB7-3

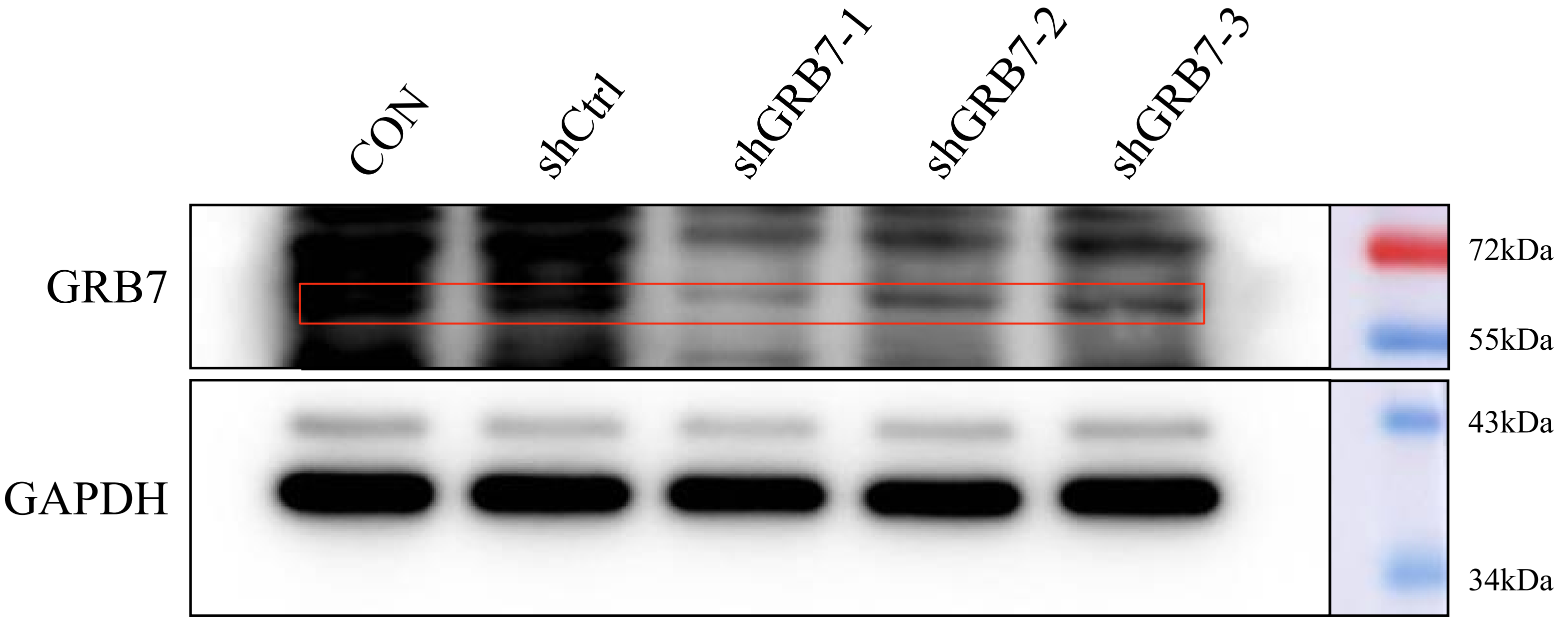

CON
shCtrl
shGRB7-1
shGRB7-2
shGRB7-3
GRB7
72kDa
55kDa
43kDa
GAPDH
34kDa

Figure 2E

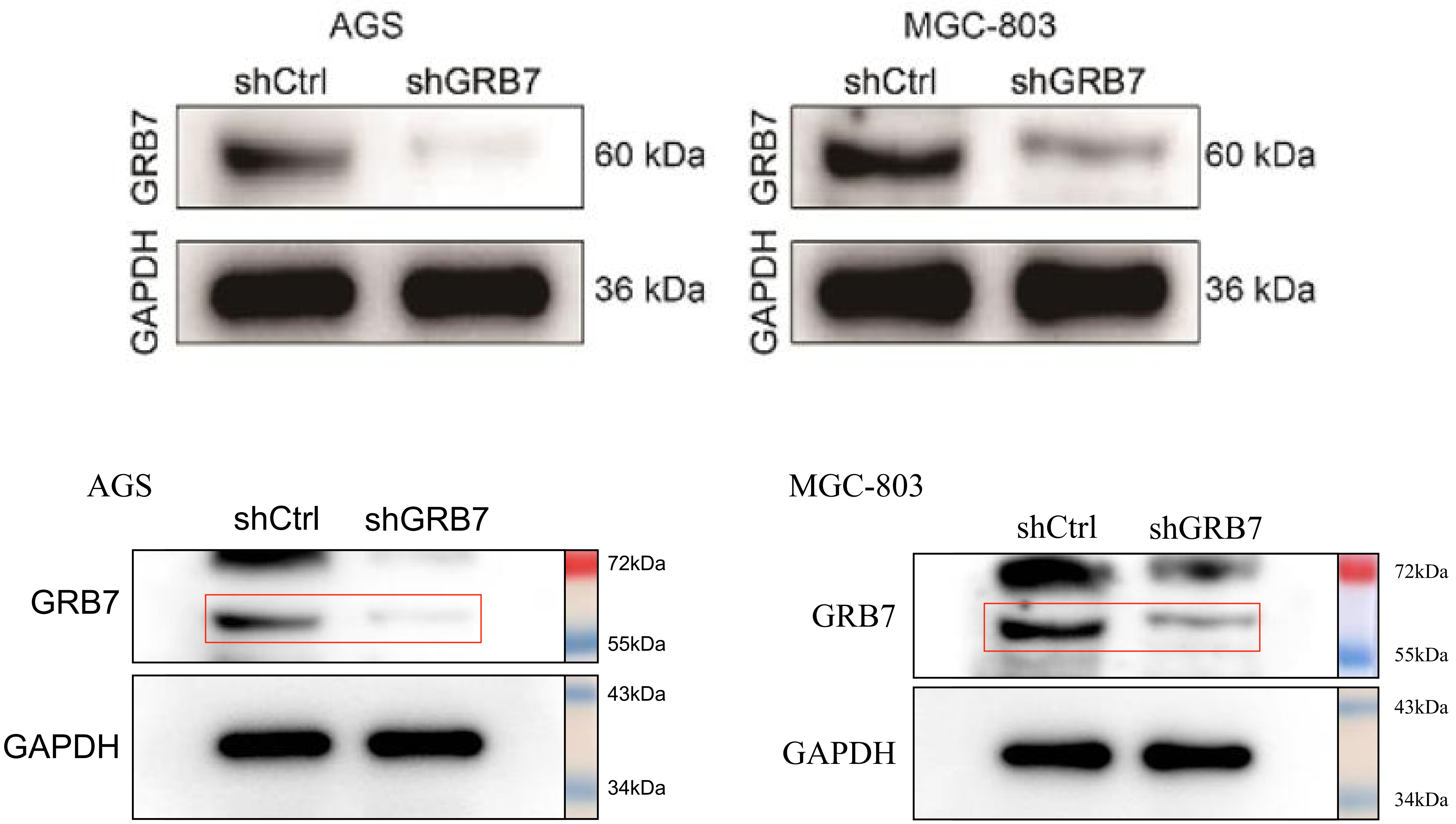

AGS
shCtrl
shGRB7
GRB7
60 kDa
GAPDH
36 kDa
MGC-803
shCtrl
shGRB7
GRB7
60 kDa
GAPDH
36 kDa
AGS
shCtrl
shGRB7
GRB7
72kDa
55kDa
GAPDH
43kDa
34kDa
MGC-803
shCtrl
shGRB7
GRB7
72kDa
55kDa
GAPDH
43kDa
34kDa

Figure 5A

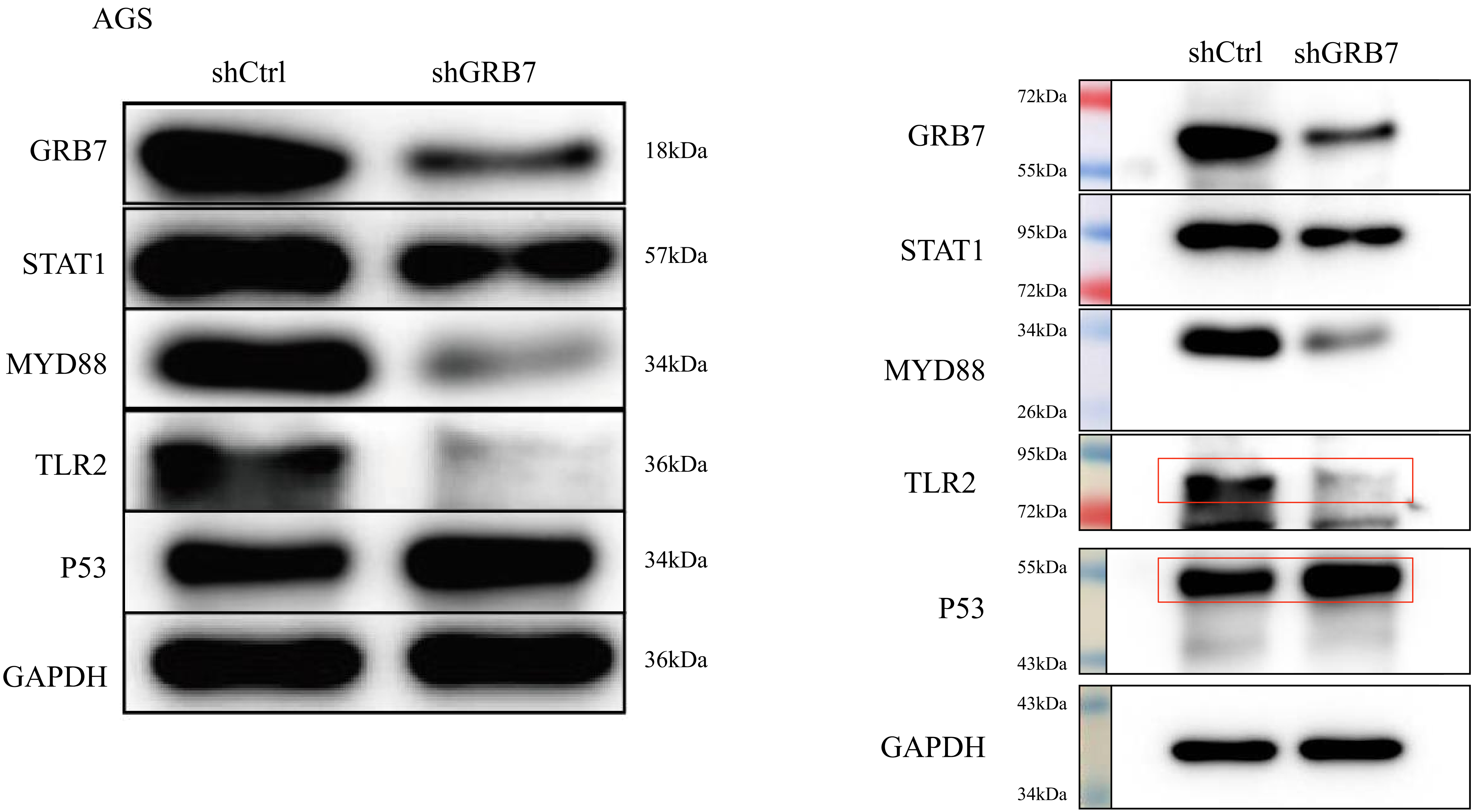
AGS
shCtrl
shGRB7
GRB7
STAT1
MYD88
TLR2
P53
GAPDH
18kDa
57kDa
34kDa
36kDa
34kDa
36kDa
shCtrl
shGRB7
GRB7
72kDa
55kDa
STAT1
95kDa
72kDa
MYD88
34kDa
26kDa
TLR2
95kDa
72kDa
P53
55kDa
43kDa
GAPDH
43kDa
34kDa

Figure 5A

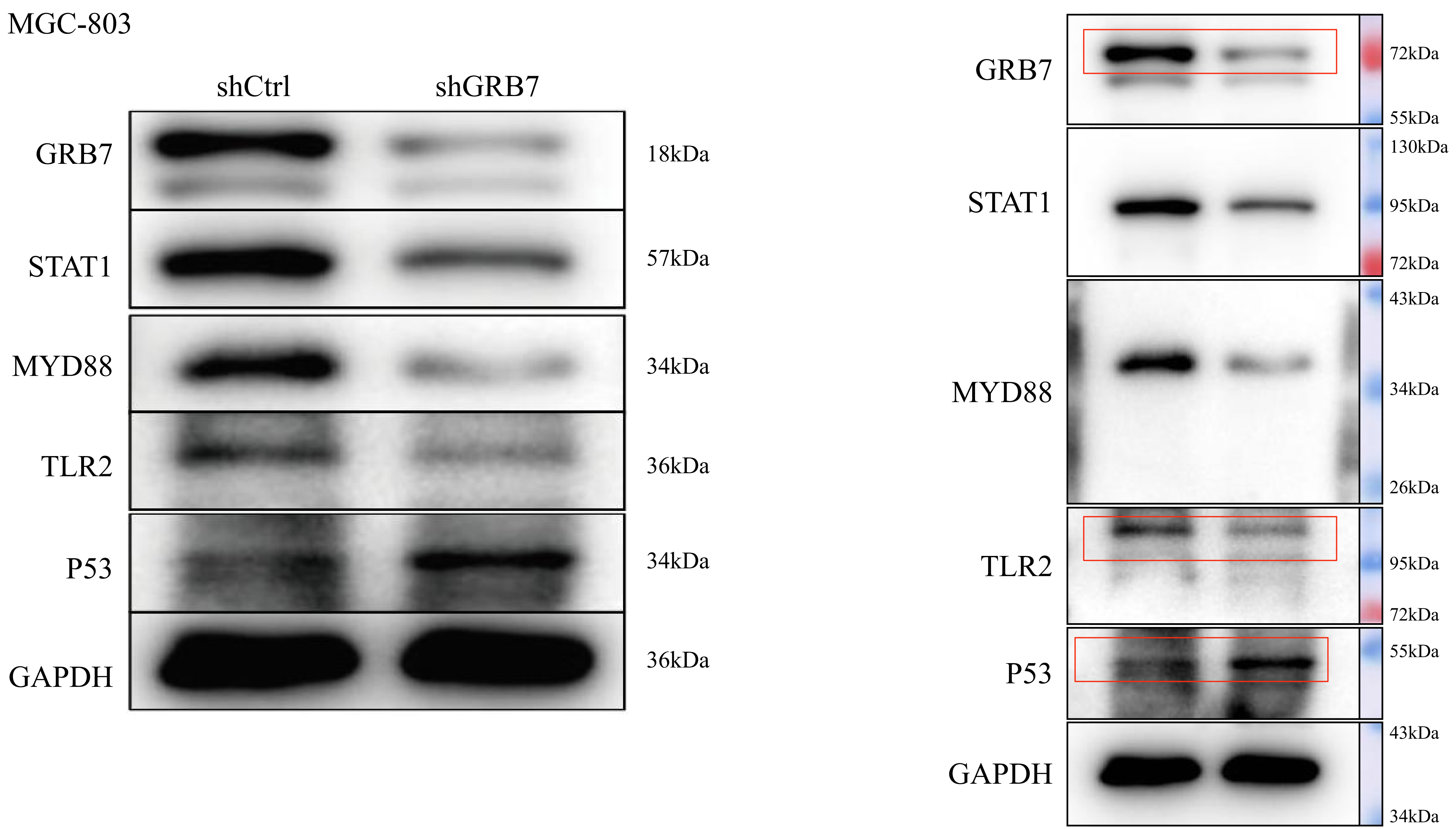
MGC-803
shCtrl
shGRB7
GRB7
STAT1
MYD88
TLR2
P53
GAPDH
18kDa
57kDa
34kDa
36kDa
34kDa
36kDa
shCtrl
shGRB7
GRB7
STAT1
MYD88
TLR2
P53
GAPDH
72kDa
55kDa
130kDa
95kDa
72kDa
43kDa
34kDa
26kDa
95kDa
72kDa
55kDa
43kDa
34kDa

Figure 2C

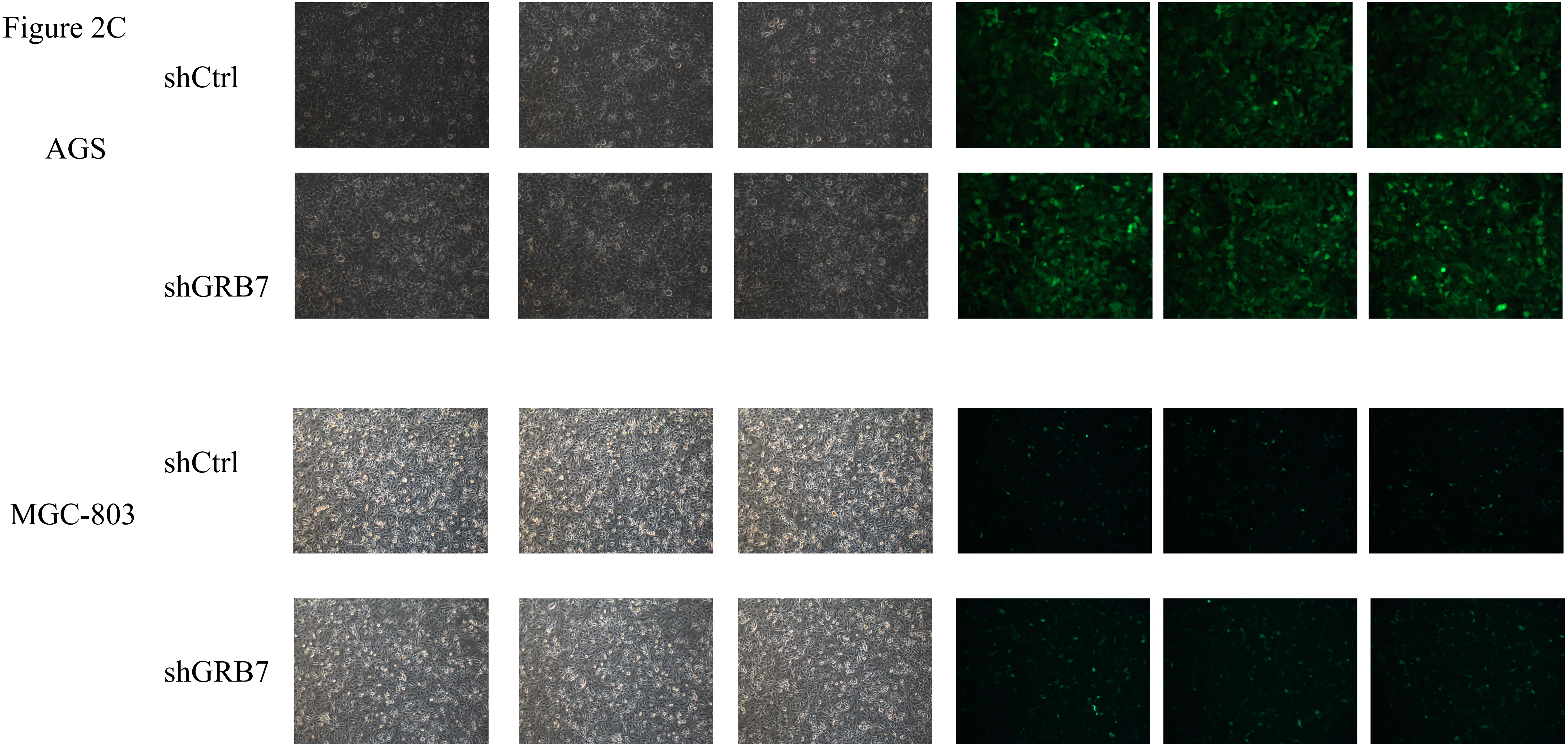

Figure 1C

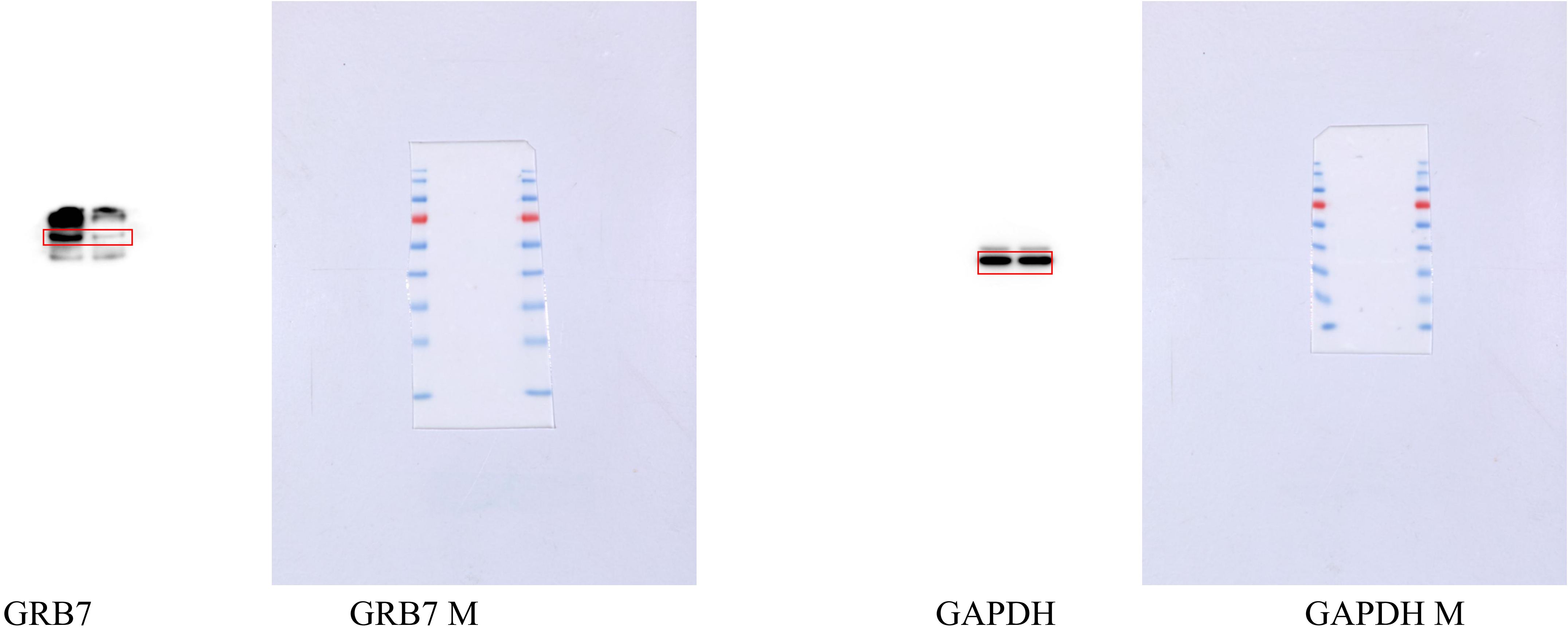

Figure 2B

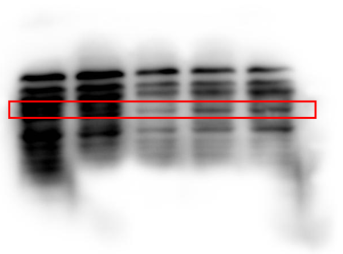

GRB7

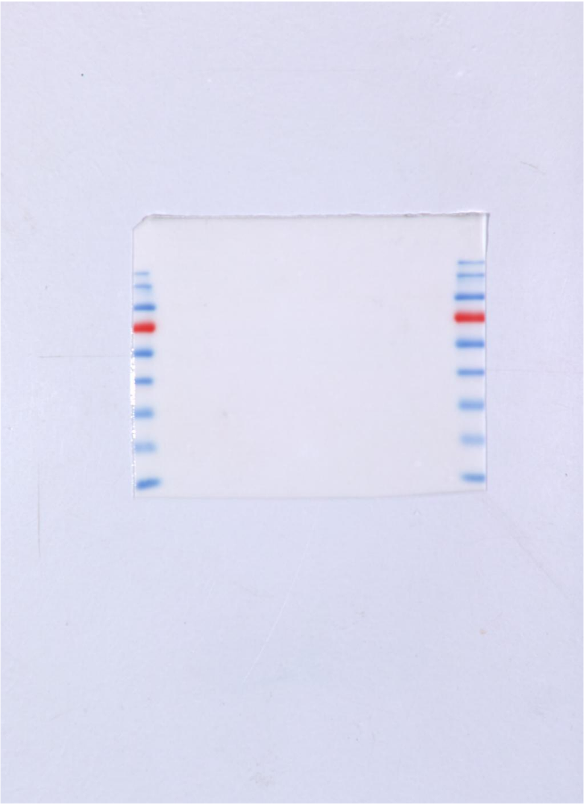

GRB7 M

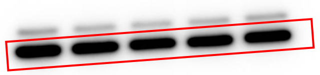

GAPDH

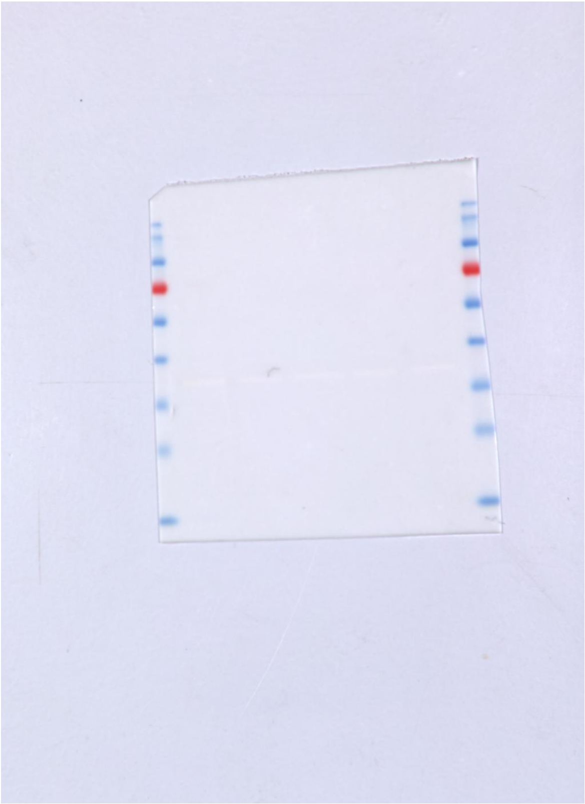

GAPDH M

Figure 2E

AGS

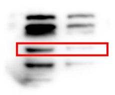

GRB7

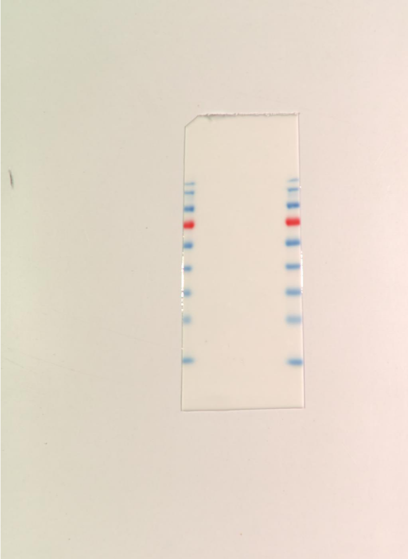

GRB7 M

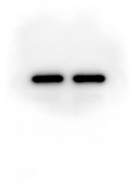

GAPDH

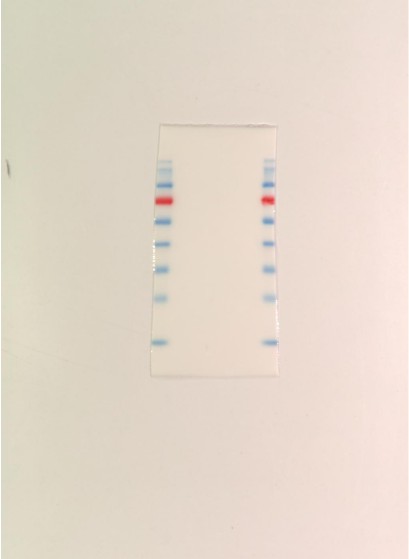

GAPDH M

MGC-803

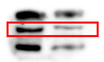

GRB7

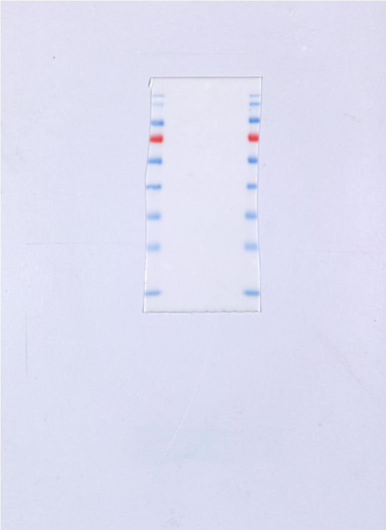

GRB7 M

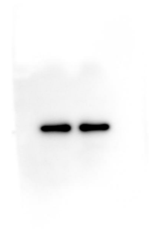

GAPDH

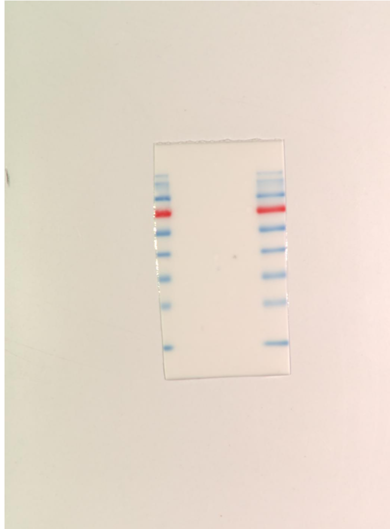

GAPDH M

Figure 5A

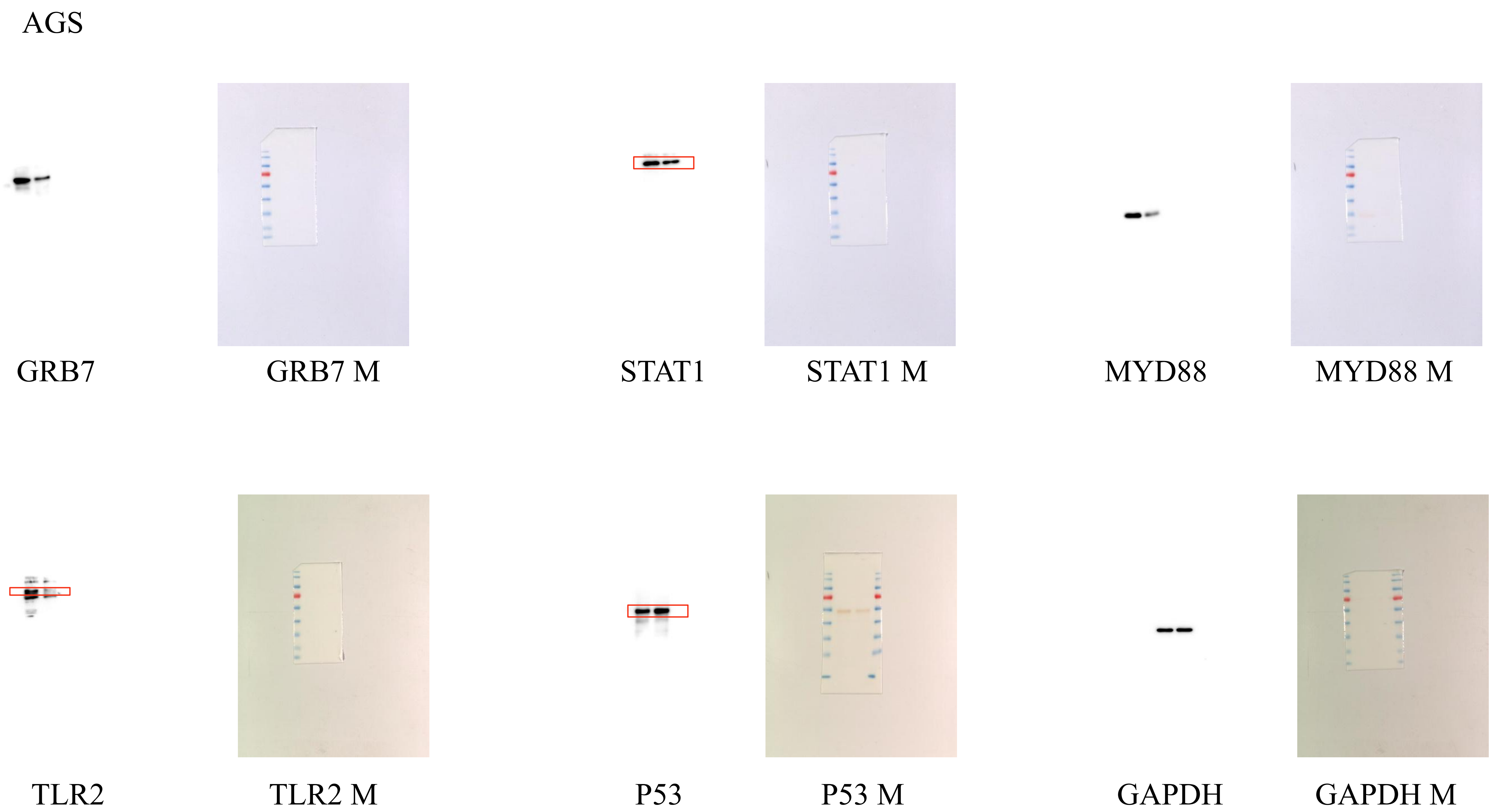
AGS
GRB7
GRB7 M
STAT1
STAT1 M
MYD88
MYD88 M
TLR2
TLR2 M
P53
P53 M
GAPDH
GAPDH M

Figure 5A

MGC-803

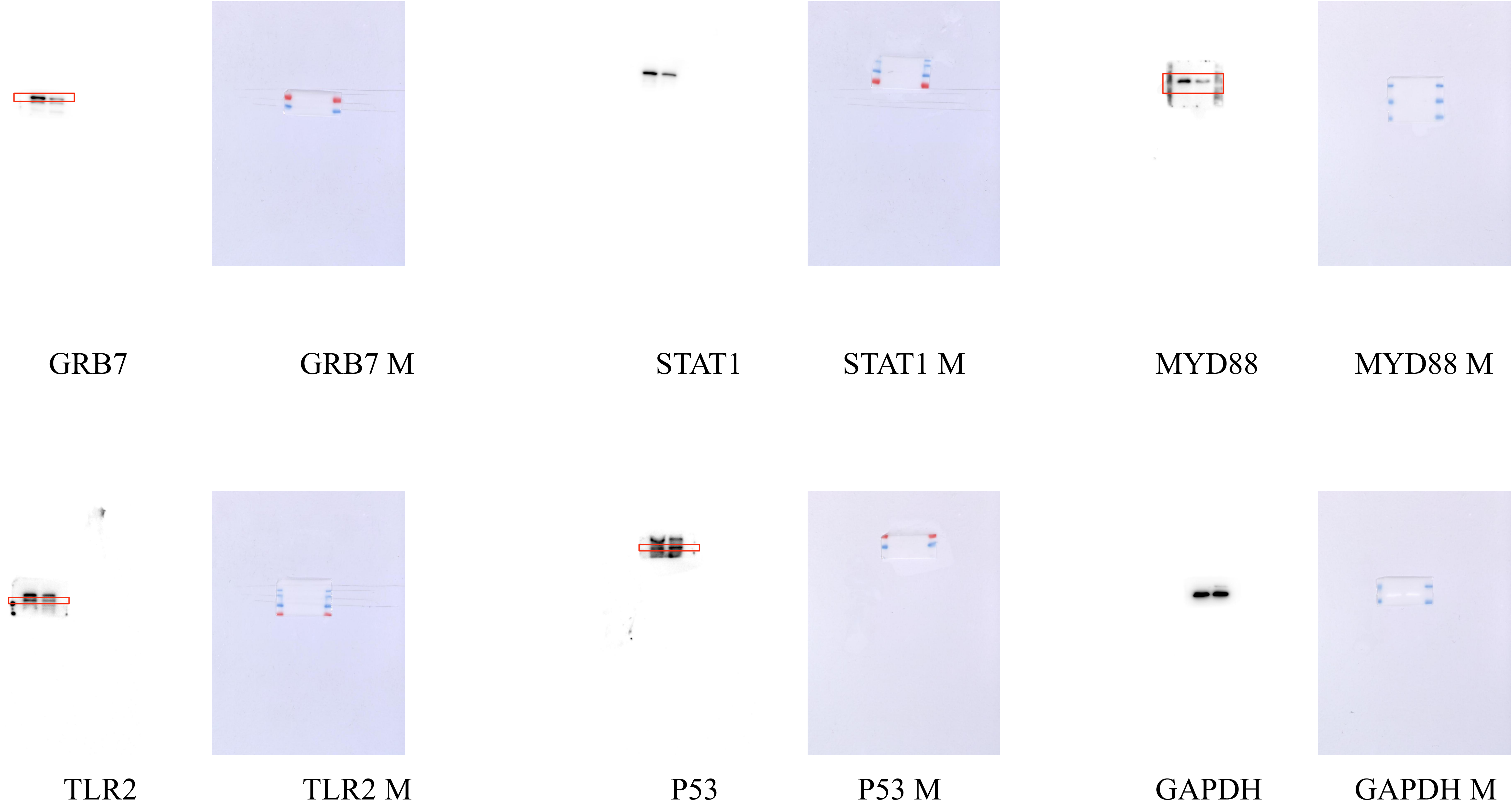

Figure 5E

Anti-GRB7

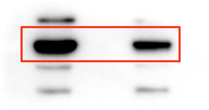

GRB7

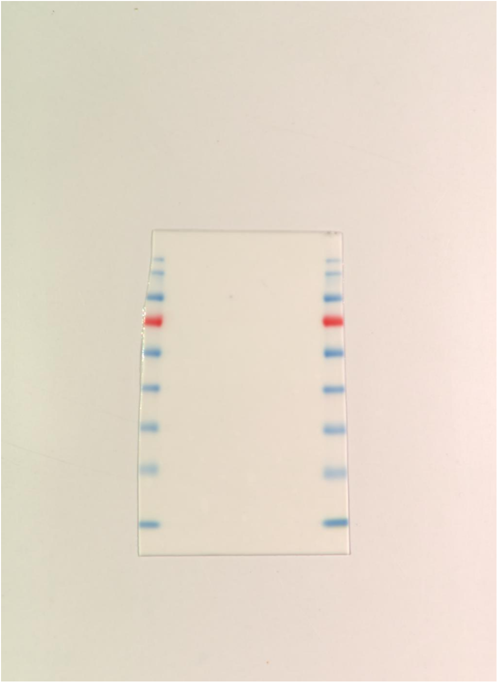

GRB7 M

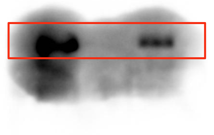

MYD88

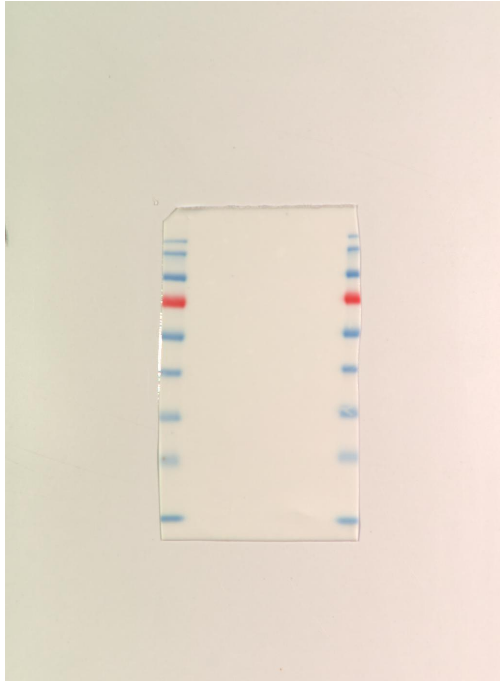

MYD88 M

Figure 5E

Anti-MYD88

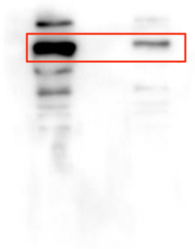

GRB7

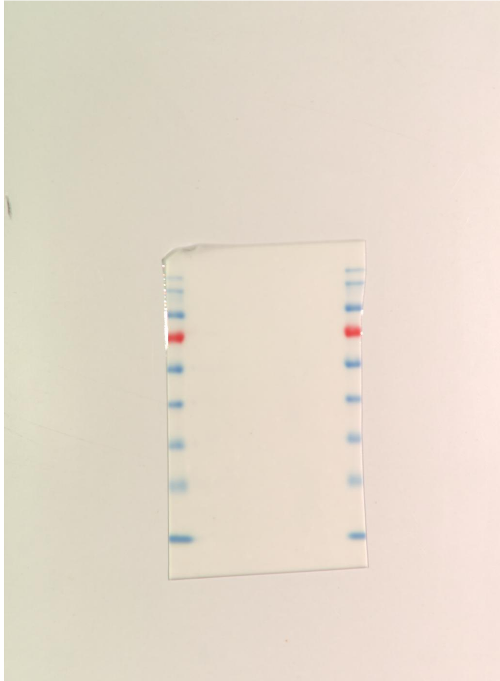

GRB7 M

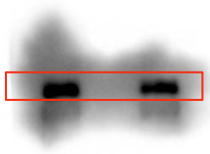

MYD88

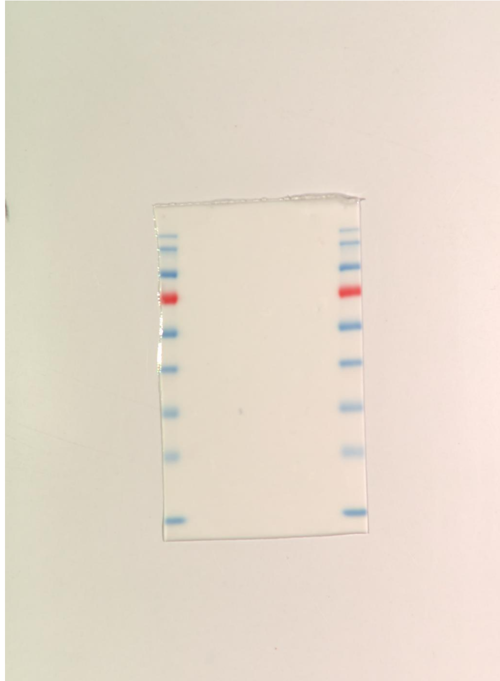

MYD88 M
